# Circular RNA circFGFR1 promotes progression and anti-PD-1 resistance by sponging miR-381-3p in non-small cell lung cancer cells

**DOI:** 10.1186/s12943-019-1111-2

**Published:** 2019-12-09

**Authors:** Peng-Fei Zhang, Xu Pei, Ke-Sang Li, Li-Na Jin, Fei Wang, Jing Wu, Xue-Mei Zhang

**Affiliations:** 1 Department of Oncology, Shanghai East Hospital, Tongji University School of Medicine, Shanghai, China; 2 Department of Cardiothoracic Surgery, the Second Affiliated Hospital of Nanchang University, Nanchang, Jiangxi China; 3 Department of Hematology and Oncology, Hwa Mei Hospital, University of Chinese Academy of Sciences, Zrhejiang, Ningbo, China; 4 Department of Hematology, Shanghai East Hospital, Tongji University School of Medicine, Shanghai, China

**Keywords:** circFGFR1, NSCLC, Immune evasion, Proliferation, Metastasis

## Abstract

**Background:**

Immune system evasion, distance tumor metastases, and increased cell proliferation are the main reasons for the progression of non-small cell lung cancer (NSCLC) and the death of NSCLC patients. Dysregulation of circular RNAs plays a critical role in the progression of NSCLC; therefore, further understanding the biological mechanisms of abnormally expressed circRNAs is critical to discovering novel, promising therapeutic targets for NSCLC treatment.

**Methods:**

The expression of circular RNA fibroblast growth factor receptor 1 (circFGFR1) in NSCLC tissues, paired nontumor tissues, and cell lines was detected by RT-qPCR. The role of circFGFR1 in NSCLC progression was assessed both in vitro by CCK-8, clonal formation, wound healing, and Matrigel Transwell assays and in vivo by a subcutaneous tumor mouse assay. In vivo circRNA precipitation, RNA immunoprecipitation, and luciferase reporter assays were performed to explore the interaction between circFGFR1 and miR-381-3p.

**Results:**

Here, we report that circFGFR1 is upregulated in NSCLC tissues, and circFGFR1 expression is associated with deleterious clinicopathological characteristics and poor prognoses for NSCLC patients. Forced circFGFR1 expression promoted the migration, invasion, proliferation, and immune evasion of NSCLC cells. Mechanistically, circFGFR1 could directly interact with miR-381-3p and subsequently act as a miRNA sponge to upregulate the expression of the miR-381-3p target gene C-X-C motif chemokine receptor 4 (CXCR4), which promoted NSCLC progression and resistance to anti-programmed cell death 1 (PD-1)- based therapy.

**Conclusion:**

Taken together, our results suggest the critical role of circFGFR1 in the proliferation, migration, invasion, and immune evasion abilities of NSCLC cells and provide a new perspective on circRNAs during NSCLC progression.

## Introduction

Lung cancer is the leading cause of cancer-related deaths worldwide [1]. The major pathological type of lung cancer is non-small-cell lung cancer (NSCLC), and NSCLC is the leading cause of cancer-related deaths in China [2]. Recently, long-term clinical benefits have been observed with immune checkpoint inhibitors, including PD-1 antibody, in patients with NSCLC; however, the majority of patients inevitably acquired resistance after several cycles of therapy [3, 4]. Thus, further understanding the molecular mechanisms that contribute to NSCLC progression is critical for developing effective therapy methods.

Circular RNAs (circRNAs) are a class of noncoding RNAs or protein-coding RNAs derived from a single exon or multiple exons and are located in the cytoplasm [5, 6]. Accumulating evidence has suggested that circRNAs can participate in several physiological and pathological processes, including tumorigenesis and cancer progression [7–9]. For example, our previous study indicated that forced circFGFR3 expression promoted NSCLC cell invasion and proliferation by competitively combining with miR-22-3p to facilitate galectin-1 action [10]. In addition, we have reported that circTRIM33–12 inhibits hepatocellular carcinoma proliferation, invasion, and immune system evasion by inhibiting oncogenic miR-191 and promoting tet methylcytosine dioxygenase 1 (TET1) expression [7]. Zhao, et al. verified that the virus-derived protein-encoding circular RNA circE7 is biologically functional and linked to the transforming properties of some human papillomaviruses (HPVs) [6]. Although the molecular mechanisms of dysregulated circRNA-related pathways have been extensively investigated, the circRNA-activated oncogenic pathways that participate in NSCLC progression are still poorly understood.

Fibroblast growth factor receptor 1 (FGFR1) is an oncogene that can promote tumor cell progression and tumorigenesis through different mechanisms in several cancers, including NSCLC [11–14]. A previous study reported that the upregulation of FGFR1 in NSCLC was caused by gene amplification [15–19], which indicated that FGFR1-derived circRNA expression might be upregulated in NSCLC. Therefore, we speculate that FGFR1-derived circRNAs may act as tumor promotors in NSCLC. In this study, we analyzed FGFR1-derived circRNA expression profiles in human NSCLC tissues and identified circFGFR1 (hsa_circ_0084003) as a significantly upregulated circRNA in NSCLC tissues. Furthermore, the expression of circFGFR1 was closely related to poor prognoses for NSCLC patients. Additionally, we found that circFGFR1 could directly interact with miR-381-3p, which subsequently acted as a miRNA sponge to upregulate the expression of the miR-381-3p target gene C-X-C motif chemokine receptor 4 (CXCR4), thereby promoting the progression of NSCLC. Thus, circFGFR1 might act as a promising therapeutic target for NSCLC therapy.

## Methods

### Cell lines and clinical tissues

The seven human NSCLC cell lines (NCI-H358, NCI-H1299, A549, HCC827, NCI-H1650, NCI-H838, and NCI-H292), the LLC mouse lung cancer cell line, and the HEK-293 T cell line were purchased from the Shanghai Institute of Cell Biology, Chinese Academy of Sciences (Shanghai, China). The cells were cultured in Dulbecco’s modified Eagle’s medium (DMEM, HyClone, Logan City, UT) supplemented with 10% fetal bovine serum (FBS, Gibco, Carlsbad, CA, USA), 100 units/ml penicillin and 100 μg/ml streptomycin (Gibco, Carlsbad, CA, USA) in a humidified atmosphere containing 5% CO_2_ at 37 °C.

### Patients and follow-up

A total of 210 NSCLC tissues and matched nontumor tissues were collected to construct the tissue microarray (TMA), which was analyzed by immunohistochemistry (IHC) and quantitative real-time polymerase chain reaction (RT-qPCR). All patients underwent curative resection, as confirmed by pathological examination at East Hospital of Tongji University, the Second Affiliated Hospital of Nanchang University, and Hwa Mei Hospital of University of Chinese Academy of Sciences. Clinicopathological information was collected from 1 January 2010 to 31 December 2013. The Ethics Committee of the East Hospital of Tongji University Biomedical Research Department provided ethical approval, and informed consent for collecting and preserving samples and documenting details was obtained from every patient.

### Quantitative real-time polymerase chain reaction (RT-qPCR), western blotting, IHC, and fluorescence in situ hybridization (FISH) assays

RT-qPCR, western blotting analysis, IHC, and FISH assays were performed as described in previous studies [7, 20] and described in Additional file 1: Supplementary Materials and Methods. The RT-qPCR primers used in this study are listed in Additional file 2: Table S1. The antibodies used in this study are listed in Additional file 3: Table S2.

### Cell proliferation, clonal formation, wound-healing cell migration, and Matrigel invasion assays

Cell proliferation, clonal formation, wound-healing cell migration, and Matrigel invasion assays were performed as described in our previous studies [7, 21] and in Additional file 1: Supplementary Materials and Methods.

### In vivo circRNA precipitation, RNA immunoprecipitation (RIP), and luciferase reporter assays

In vivo circRNA precipitation (circRIP), RIP, and luciferase reporter assays were performed as described in previous studies [7, 20, 22]. Biotin-labeled circFGFR1 and negative control probes were synthesized by the GeneChem Company. In brief, A549 cells were washed with cold phosphate-buffered saline, fixed with 1% formaldehyde, lysed in co-IP buffer, sonicated and centrifuged. Then, the supernatant was cultured with M280 streptavidin Dynabeads (Invitrogen) mixture and incubated at 30 °C for 12 h. Subsequently, the mixture was washed and incubated with lysis buffer and proteinase K. RNA was extracted from the mixture using TRIzol Reagent (Invitrogen).

The RIP assay was carried out using a Magna RIP RNA-binding protein immunoprecipitation kit (Millipore). In brief, cell lysates were cultured with Dynabeads coated with AGO2 antibody or IgG antibody at 4 °C for 12 h, and total RNA was extracted for the detection of enriched circFGFR1 and miRNA by RT-qPCR.

For the luciferase reporter assay, potential binding sites were predicted using StarBase v3.0. HEK-293 T cells were cotransfected with pGL3-LUC-circFGFR1, pGL3-LUC-UBE2C/FGF7/FGFR2/FGFR1/IGF2BP1/MET/CXCR4 3′ UTR or mutant pGL3-LUC-circNT5E, pGL3-LUC-UBE2C/FGF7/FGFR2/FGFR1/IGF2BP1/MET/CXCR4 3′ UTR and miR-381-3p mimics or negative control mimics. Forty-eight hours later, the cells were harvested, and the luciferase activity was measured with the dual-luciferase reporter assay system (Promega). The argonaute 2 (AGO2) and IgG antibodies used in this study are listed in Additional file 3: Table S2.

### Knocked down or overexpressed circFGFR1 transfection experiment

Small hairpin RNAs (shRNAs) targeting the junction region of the circFGFR1 sequence and circFGFR1-overexpressing lentivirus were synthesized by Hanbio Company (Shanghai, China). NSCLC cell lines were transfected with circFGFR1 shRNA or the circFGFR1-overexpressing lentivirus according to the manufacturer’s instructions.

### In vivo tumor growth and metastasis assays

In vivo tumor growth and metastasis experiments in nude mice were approved by the Animal Experimentation Ethics Committee of East Hospital, Tongji University. The experiments were performed as described in our previous studies [21, 23] and in Additional file 1: Supplementary Materials and Methods.

### Establishment of stable CXCR4-knockout cells

CRISPR/Cas9 double-vector lentivirus was used to establish stable CXCR4-knockout NSCLC cell lines. Double-vector lentivirus was purchased from the GeneChem Company and transfected as described in reference [24].

### Mice xenograft anti-PD-1 therapy study

The experiments in the C57BL/6 mice were approved by the Animal Experimentation Ethics Committee of East Hospital, Tongji University. A total of 2 × 10^6^ cells (LLC with or without increased circFGFR1 expression) were implanted subcutaneously in the left flank of the six-week-old C57BL/6 mice. When tumors reached a size of approximately 100 mm^3^, the mice were randomly assigned to 4 groups. Then, the mice were injected in the tail vein with a PD-1 monoclonal IgG antibody (a gift from Hengrui Medicine Company) or its mouse isotype control at 100 μg per dose three times a week for two weeks. Animals were euthanized when tumors reached a maximum of 1000 mm^3^ (*n* = 6). The day that the mice received the first therapy is considered day 1.

### Statistical analysis

Statistical analysis was performed with SPSS software (19.0; SPSS, Inc., Chicago, IL) as in our previous study [7] and described in Additional file 1: Supplementary Materials and Methods. *P* < 0.05 was considered statistically significant.

## Results

### Identification of FGFR1-derived circRNAs in the NSCLC cells

**Fig. 1:**
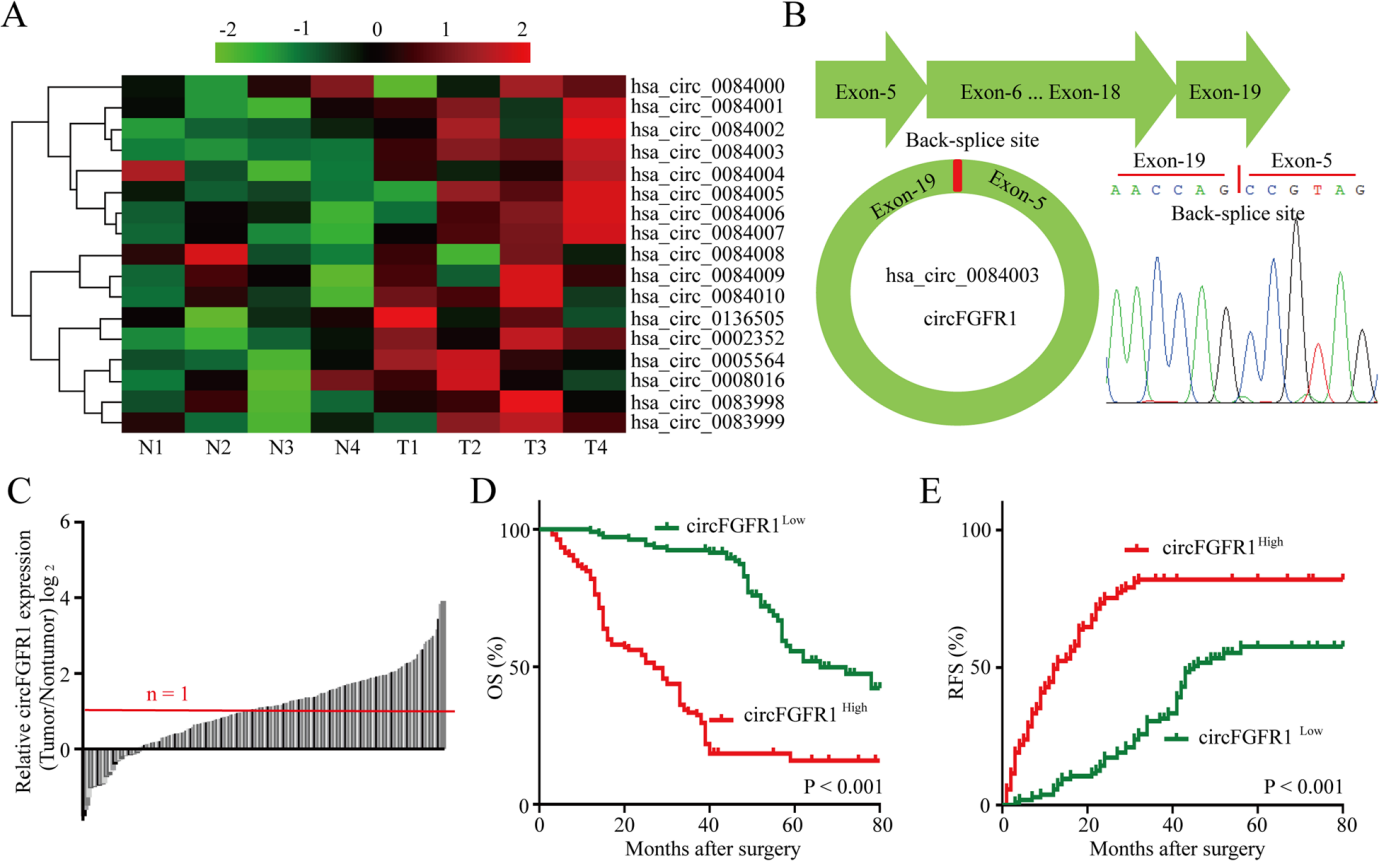
High circFGFR1 expression in the NSCLC tissues and prognostic significance. **a** The heatmap shows circRNAs derived from the FGFR1 gene in NSCLC tissues compared with those in adjacent nontumor tissues, as analyzed by RT-qPCR. **b** The scheme illustrating the production of circFGFR1. **c** The differential expression of circFGFR1 in the NSCLC tissues and adjacent nontumor tissues of 210 patients, as indicated. **d** and **e** Prognostic analysis of circFGFR1 expression in 210 NSCLC patients

In terms of the critical roles of the FGFR1-related pathway in NSCLC progression, FGFR1 amplification has been identified in all types of lung carcinoma [25–27]. Therefore, we examined 17 circular RNAs derived from FGFR1 genes by analyzing the circular RNA sequencing data from circBase. Among 17 circRNAs, we found that circFGFR1 (hsa_circ_0084003) expression levels were significantly and consistently upregulated in four of the NSCLC tumor tissues compared to the expression levels in the matched adjacent nontumor lung tissues (Fig. 1a). CircFGFR1 was composed of 1844 nucleotides and 13 exons (Fig. 1b). To explore the relationship between circFGFR1 and clinical characteristics, we measured circFGFR1 expression in the NSCLC tissues and adjacent nontumor tissues and found that circFGFR1 was significantly upregulated in the NSCLC tissues (111/210) (Fig. 1c). Next, we explored the relationship between circFGFR1 expression and the clinicopathological characteristics of 210 NSCLC patients, as listed in Table 1. The results showed that NSCLC patients with circFGFR1^high^ cells had larger tumors (*P* = 0.010), lymph node metastasis (*P* = 0.004), and poor cell differentiation (*P* = 0.019). Then, we explored the prognostic implications of circFGFR1 expression. Importantly, our results showed that patients with circFGFR1^high^ cells expression had a significantly worse prognosis than those with circFGFR1^low^ (Fig. 1d and e). The results from the multivariate analysis indicated that circFGFR1 expression is an independent predictor for postoperative recurrence and overall survival (OS; Tables 2 and Table 3). These results indicate that circFGFR1 likely participates in the progression of NSCLC.

**Table 1:** Correlation between circFGFR1 and clinical characteristics in 210 NSCLC patients

Variables	CircFGFR1 expression level		*P* value
	Low	High	
Age			
60 <	52	65	0.095
60 ≥	53	40	
Gender			
Male	74	66	0.306
Female	31	39	
Smoking status			
Smokers	69	73	0.658
Nonsmokers	36	32	
Histological type			
Squamous cell carcinoma	24	31	0.346
Adenocarcinomas	81	74	
Tumor stage			
I–II	44	53	0.268
III–IV	61	52	
Lymph node metastasis			
Yes	37	61	0.010
No	68	44	
Tumor size			
≤ 3 cm	37	59	0.004
> 3 cm	68	46	
Differentiation			
Well and moderate	42	60	0.019
Poor	63	45

**Table 2:** Univariate and Multivariate Analyses of Factors Associated with Overall Survival

Factors	OS			
	Univariate, *P*	Multivariate		
		HR	95% CI	*P* value
Sex (Female vs. Male)	0.372			NA
Age (years) (≤ 60 vs. > 60)	0.821			NA
Smoking status (Smokers vs. Nonsmokers)	0.117			NA
Histological type (SCC vs. Adenocarcinomas)	0.061			NA
Lymph node metastasis (Yes vs. No)	0.026			NS
Differentiation (Well and moderate vs. Poor)	0.014			NS
Tumor size (diameter, cm) (> 3 vs. ≤ 3)	0.007	1.417	1.094–2.217	0.031
TNM (III-IV vs. I-II)	0.056			NA
CircFGFR1 expression (High vs. Low)	0.002	1.574	0.911–1.927	0.013

*OS* Overall survival, *NA* Not adopted, *NS* Not significantly, *SCC* Squamous cell carcinoma, *95%CI* 95% confidence interval, *HR* Hazard ratio; Cox proportional hazards regression model

**Table 3:** Univariate and Multivariate Analyses of Factors Associated with Cumulative Recurrence

Factors	Cumulative Recurrence			
	Univariate, *P*	Multivariate		
		HR	95% CI	*P* value
Sex (Female vs. Male)	0.414			NA
Age (years) (≤ 60 vs. > 60)	0.871			NA
Smoking status (Smokers vs. Nonsmokers)	0.332			NA
Histological type (SCC vs. Adenocarcinomas)	0.087			NA
Lymph node metastasis (Yes vs. No)	0.074			NA
Differentiation (Well and moderate vs. Poor)	0.062			NA
Tumor size (diameter, cm) (> 3 vs. ≤ 3)	0.037			NS
TNM (III-IV vs. I-II)	0.016			NS
CircFGFR1 expression (High vs. Low)	0.006	1.443	1.077–2.824	0.023

*NA* Not adopted, *NS* Not significantly, *SCC* Squamous cell carcinoma, *95%CI* 95% confidence interval, *HR* hazard ratio; Cox proportional hazards regression model

### CircFGFR1 promotes NSCLC cell proliferation, migration, and invasion in vitro

**Fig. 2:**
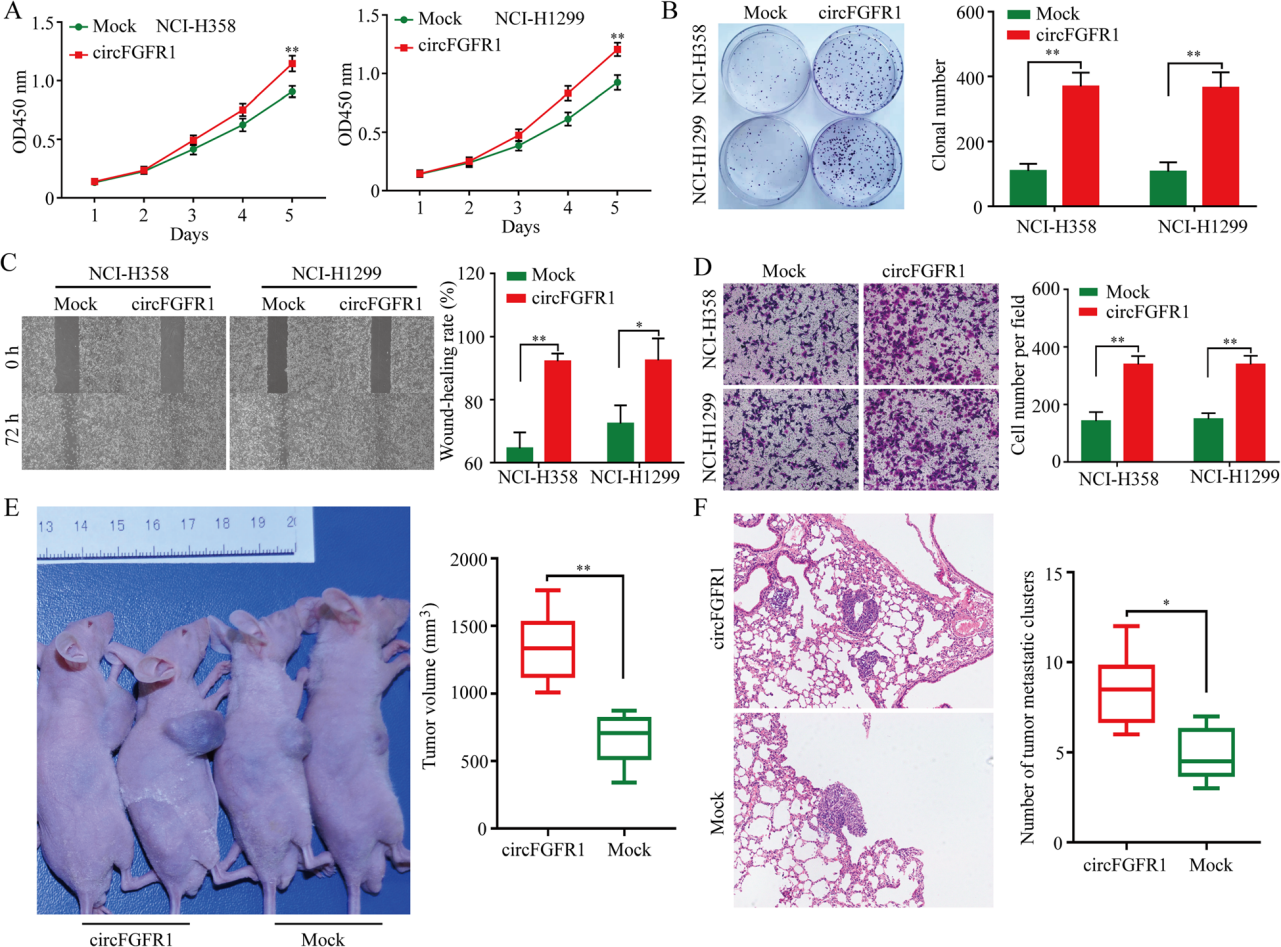
Effects of forced circFGFR1 expression on the progression of the NSCLC cells. **a** and **b** NSCLC cell proliferation after the expression of circFGFR1 was upregulated, as assessed by CCK-8 assay (**a**) and clonal formation assay (**b**). **c** and **d** NSCLC cell migration and invasion after the expression of circFGFR1 was upregulated, as assessed by wound-healing assay (**c**) and Matrigel Transwell assay (**d**). **e** and **f** Tumor growth and metastatic ability of NSCLC cells with the upregulated circFGFR1 expression was investigated in nude mice xenograft tumor models. The data are presented as the mean ± SD, *P < 0.05, **P < 0.01

To explore the biological functions of circFGFR1 in NSCLC, we measured circFGFR1 expression in seven types of human NSCLC cells (Additional file 4: Figure S1a). Next, we designed two shRNA plasmids to target the unique back-splice junction. The back-splice junction-specific shRNA (shcircF1 and shcircF2) effectively knocked down circFGFR1 expression but had no effect on the level of FGFR1 mRNA in the A549 and HCC827 cells (cell lines with high circFGFR1 expression) (Additional file 4: Figure S1b-c). Using the above-mentioned vector, we succeeded in overexpressing circFGFR1 in NCI-H358 and NCI-H1299 cells (Additional file 4: Figure S1d). In vitro CCK-8, clone formation, wound-healing cell migration, and invasion assays revealed that the NCI-H358 and NCI-H1299 cells (which had low circFGFR1 expression) in which circFGFR1 expression was forced were significantly more likely to exhibit a malignant phenotype than the mock cells (Fig. 2a-d). Conversely, reduced circFGFR1 expression inhibited the proliferation, migration, and invasion abilities of the A549 and HCC827 cells, according to the results from the CCK-8, clonal formation, wound healing, and Matrigel Transwell assays (Additional file 4: Figure S2a-d). To verify the in vitro findings, we examined the biological role of circFGFR1 in mediating in vivo proliferation. NCI-H358 cancer cells with stably forced circFGFR1 expression were subcutaneously implanted into nude mice. Consistent with the above in vitro findings, the overexpression of circFGFR1 dramatically promoted tumor growth and lung metastasis (Fig. 2e and f).

### CircFGFR1 serves as a sponge for multiple miRNAs

**Fig. 3:**
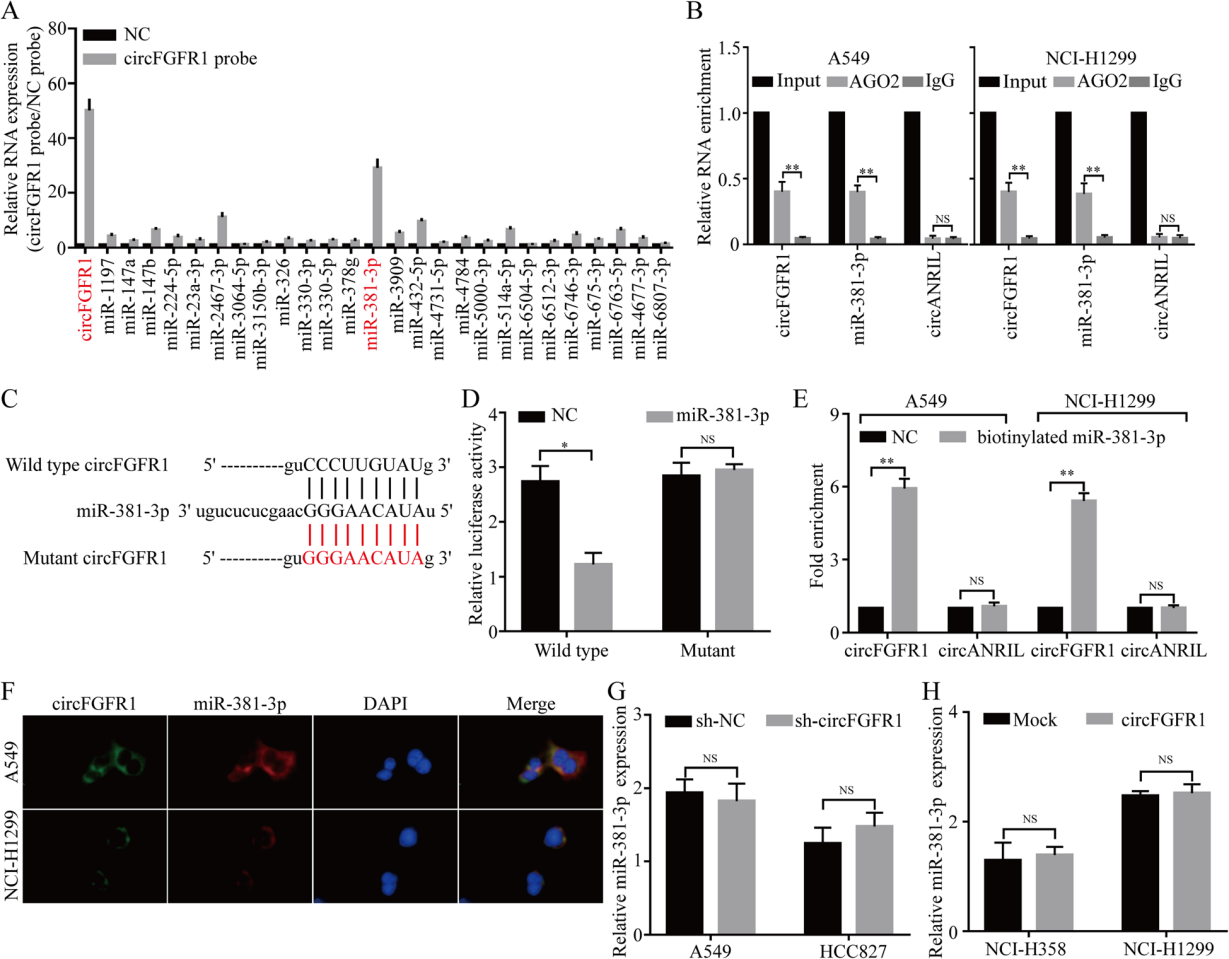
CircFGFR1 may function as a sponge for miR-381-3p. **a** RIP was performed for circRNA in A549 cells using a circFGFR1 probe and a negative control (NC) probe. **b** RIP experiments were carried out using an AGO2 antibody with NSCLC cell extracts. **c** Putative binding sites of miR-381-3p with respect to circFGFR1 were predicated via StarBase v3.0. **d** The luciferase activity of pLG3-circFGFR1 in the HEK-293 T cells after cotransfection with miR-381-3p. **e** The level of circFGFR1 in the streptavidin-captured fractions of the NSCLC cell lysates after transfection with biotinylated miR-381-3p or the negative control (NC). CircANRIL was used as a negative control. **f** miR-381-3p and circFGFR1 were detected by RNA in situ hybridization in the NSCLC cells. **g** and **h** The relative level of miR-383-3p was measured by RT-qPCR in the NSCLC cells transfected with circFGFR1, shcircFGFR1, or the control. The data are presented as the mean ± SD, *P < 0.05, **P < 0.01

An increasing number of studies have reported that circRNAs act as miRNA sponges; therefore, we investigated whether circFGFR1 has the ability to bind to miRNAs. Through StarBase v3.0, we found that 26 miRNAs were predicated to be possible targets of circFGFR1. To verify the critical functional miRNAs that may interact with circFGFR1 in NSCLC cells, a circFGFR1-specific probe was used to perform RNA in vivo precipitation (RIP) in A549 cells, which were then screened by the RT-qPCR for the potential miRNAs that had been predicted. Using RIP circFGFR1 pull-down experiments, we purified circFGFR1-associated RNAs and analyzed 26 candidate miRNAs in the complex. The results showed a specific enrichment of circFGFR1 and miR-381-3p compared to the negative control probe, whereas the other miRNAs were slightly enriched or not enriched, indicating that miR-381-3p is one of the critical circFGFR1-associated miRNAs in NSCLC cells (Fig. 3a). Next, we performed RNA immunoprecipitation (RIP) with argonaute 2 (AGO2) antibody in A549 and NCI-H1299 cells. Our results showed that circFGFR1 and miR-381-3p, but not circANRIL (a circular RNA that reportedly does not bind to AGO2) [20], were significantly enriched, as they were precipitated by the AGO2 antibody (Fig. 3b). These results indicated that circFGFR1 may act as a binding platform for AGO2 and miR-381-3p. To verify these results, we performed a luciferase assay using miR-381-3p mimics cotransfected with luciferase reporters (which contained a wild-type or miR-381-3p-target mutant circFGFR1 sequence) into HEK-293 T cells. Compared with the negative control RNA (NC), miR-381-3p decreased the luciferase reporter activity significantly in the cells with the wild-type circFGFR1 sequence but not the cells with either the wild-type- or the miR-381-3p-target mutant circFGFR1 sequence (Fig. 3c and d). Furthermore, using a pull-down assay with biotinylated miR-381-3p mimics, we observed significant enrichment of circFGFR1 compared with the level in the negative controls, while circANRIL was not enriched in the A549 and the NCI-H1299 cells (Fig. 3e). Moreover, the results from the double FISH assay indicated the colocalization of circFGFR1 and miR-381-3p in the NSCLC cells (Fig. 3f). In addition, miR-381-3p did not show significant changes after circFGFR1 was overexpressed or silenced, and circFGFR1 did not show significant changes after miR-381-3p expression was either upregulated or knocked down (Fig. 3g and h; Additional file 4: Figure S3). These findings indicate that circFGFR1 and miR-381-3p are likely not degraded by each other. All of the above experiments confirmed that circFGFR1 may function as a sponge for miR-381-3p in NSCLC cells.

### CircFGFR1 upregulates CXCR4 expression sponging miR-381-3p

**Fig. 4:**
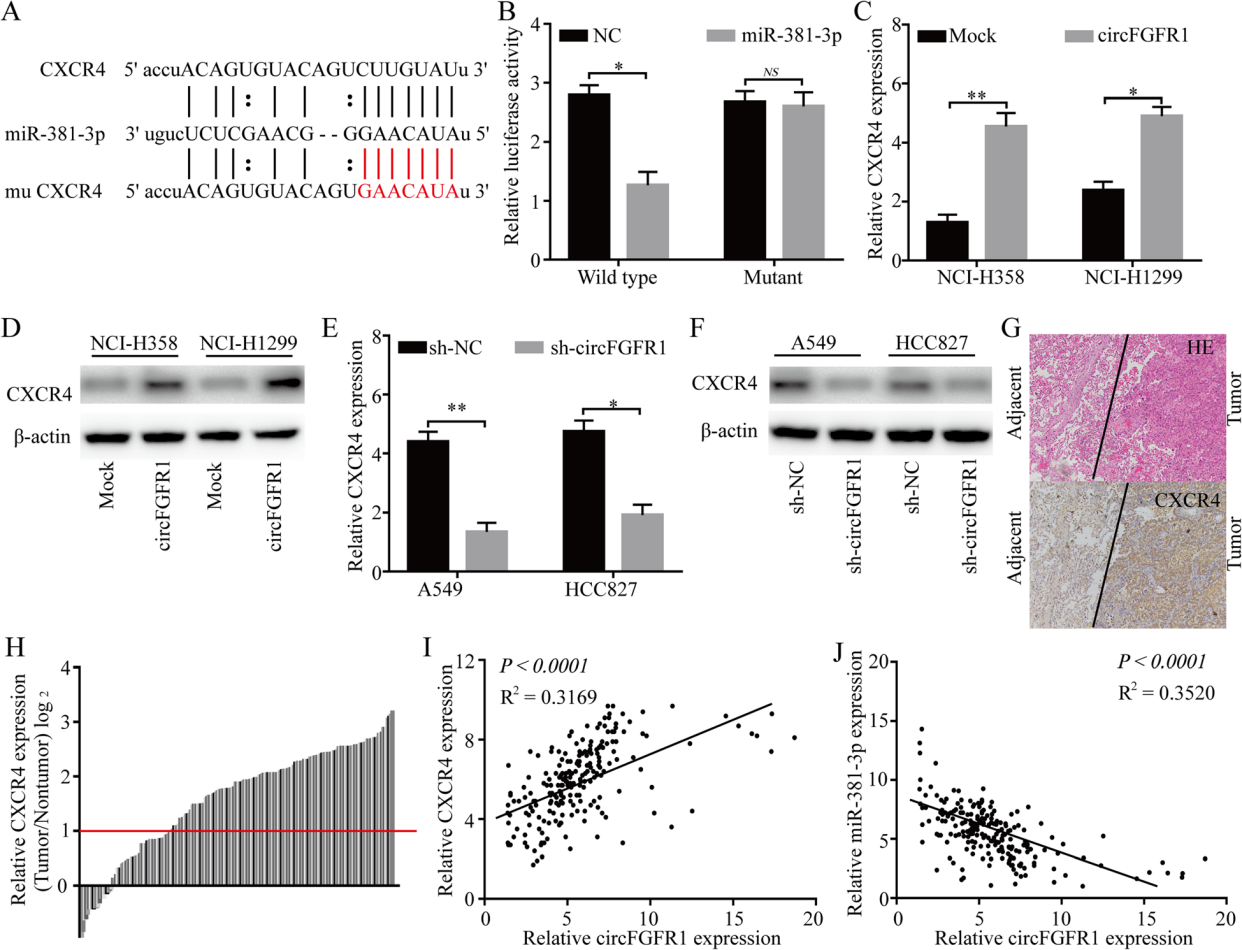
CircFGFR1 regulates the miR-381-3p/CXCR4 pathway in the NSCLC cells. **a** Putative binding sites of miR-381-3p with respect to CXCR4 were predicated via StarBase v3.0. **b** The luciferase activity of pLG3-CXCR4 in the HEK-293 T cells cotransfected with miR-381-3p. **c-f** The levels of CXCR4 mRNA and protein were measured by RT-qPCR or western blotting in the NSCLC cells transfected with circFGFR1, shcircFGFR1, or the control. **g** Representative NSCLC cases were analyzed by IHC staining for CXCR4. **h** The CXCR4 levels in 210 pairs of NSCLC and matched nontumor tissues, shown as log_2_ (tumor/nontumor). **i** A positive correlation between circFGFR1 and CXCR4 expression was observed in the NSCLC tissues (*R*^*2*^ = 0.3169; *P* < 0.0001). **j** A negative correlation between circFGFR1 and miR-381-3p expression was observed in the NSCLC tissues (*R*^*2*^ = 0.3520; *P* < 0.0001). The data are presented as the mean ± SD. NS: not significant, *P < 0.05, **P < 0.01

To further explore the function of miR-381-3p in NSCLC cells, StarBase v3.0, the PITA and miRanda algorithms were used to predict potential targets of miR-381-3p. The predicted result showed that several oncogenes, including UBE2C, FGF7, FGFR2, FGFR1, IGF2BP1, MET, and CXCR4 3′ UTR mRNAs, contained target sequences for miR-381-3p (Fig. 4a). To verify whether the 3′ UTR of UBE2C, FGF7, FGFR2, FGFR1, IGF2BP1, MET, and CXCR4 mRNAs were targets of miR-381-3p in the NSCLC cells, a pLG3 luciferase reporter gene assay was used. The wild-type (wt) 3′ UTR sequence and the mutant (mu) 3′ UTR sequence of UBE2C, FGF7, FGFR2, FGFR1, IGF2BP1, MET, and CXCR4 were cloned and placed into a pLG3 luciferase reporter vectors. The luciferase activity was significantly inhibited by the miR-381-3p mimics in the HEK-293 T cells transfected with the wt 3′ UTR sequence. The luciferase activity was not changed by the miR-381-3p mimics in the HEK-293 T cells transfected with the mu 3′ UTR sequence (Fig. 4b; Additional file 4: Figure S4a). In addition, the levels of CXCR4 mRNA and protein were significantly decreased after miR-381-3p expression was upregulated in the A549 and HCC827 cells (Additional file 4: Figure S5a and b), and the levels of CXCR4 mRNA and protein were significantly increased after miR-381-3p expression was knocked down in the NCI-H358 and NCI-H1299 cells (Additional file 4: Figure S5c and d). We also used to RT-qPCR to measure the FGF7, FGFR2, FGFR1, IGF2BP1, and UBE2C mRNA expression in the A549 cells with or without forced miR-381-3p expression. The results showed that the UBE2C/FGF7/FGFR1/IGF2BP1/MET mRNA level was slightly inhibited by miR-381-3p in the A549 cells; however, FGFR2 was not inhibited (Additional file 4: Figure S4b). Moreover, the levels of CXCR4 mRNA and protein significantly were increased after the circFGFR1 expression was upregulated in the NCI-H358 and NCI-H1299 cells (Fig. 4c and d), and the levels of CXCR4 mRNA and protein expression were significantly decreased after circFGFR1 expression was knocked down in the A549 and HCC827 cells (Fig. 4e and f). Furthermore, using IHC, we measured the expression levels of CXCR4 in the 210 NSCLC patient tissues (Fig. 4g and h). A positive relationship between circFGFR1 and CXCR4 was found in the NSCLC patient tissue samples (Fig. 4i). In addition, using RT-qPCR, we measured the expression of miR-381-3p in the 210 NSCLC patient tumor tissues. A negative relationship between circFGFR1 and miR-381-3p was found in the NSCLC patient tissues (Fig. 4j). Finally, we explored the prognostic implications of CXCR4 and miR-381-3p expression. Importantly, our results showed that patients with CXCR4^high^ or miR-381-3p^low^ cells had significantly worse prognoses than those with CXCR4^low^ or miR-381-3p^high^ cells (Additional file 4: Figure S6).

### Knocking out CXCR4 reverses circFGFR1-induced cell proliferation, migration, and invasion in the NSCLC cells

**Fig. 5:**
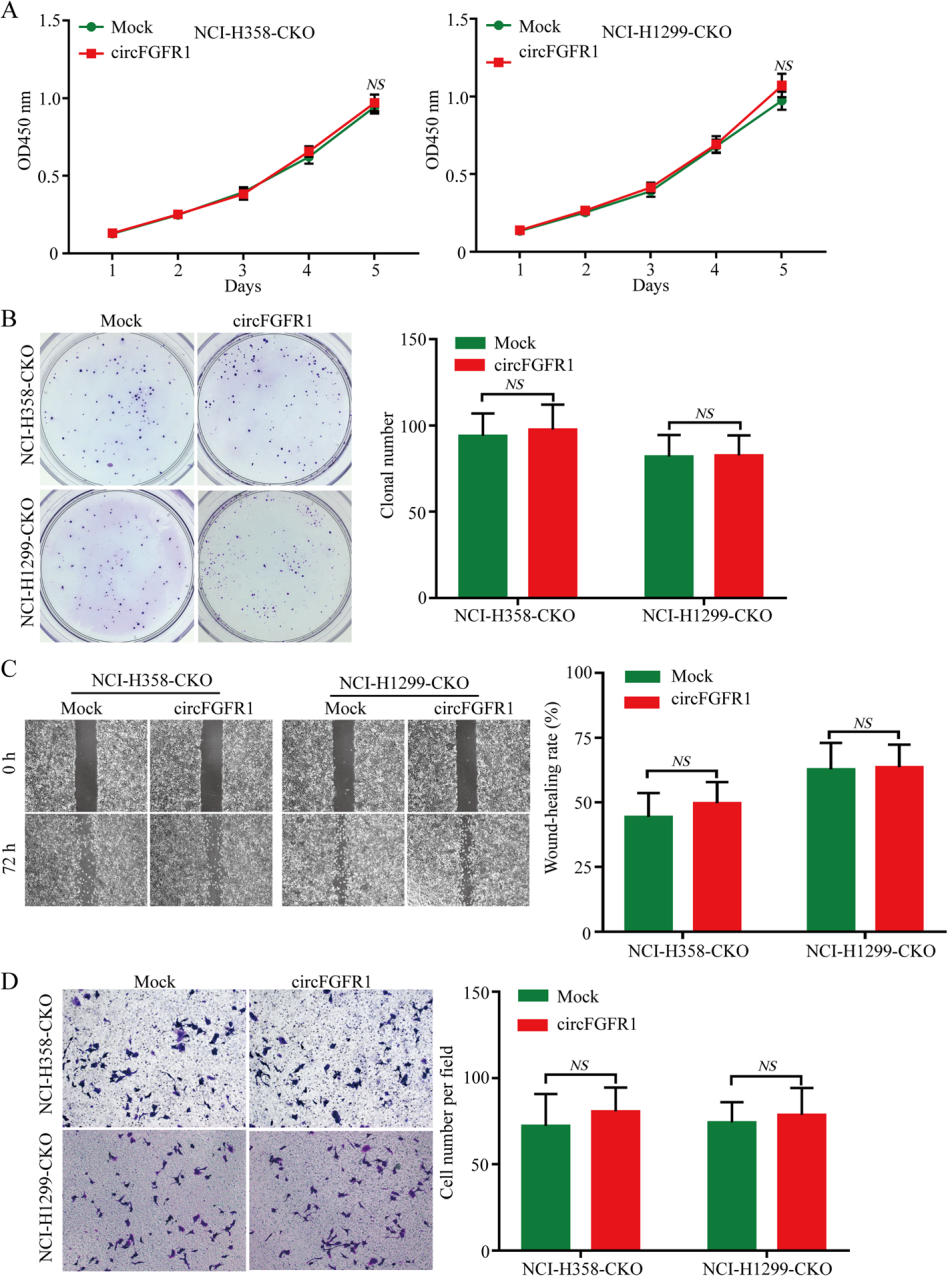
Knocking out CXCR4 reverses the oncogenic effect of circFGFR1 in the NSCLC cells in vitro. **a** and **b** Proliferation of CXCR4-knockout NSCLC cells with upregulated circFGFR1 expression was assessed by CCK-8 assay (**a**) and clonal formation assay (**b**). **c** and **d** Cell migration and invasion of the CXCR4-knockout NSCLC cells with upregulated circFGFR1 expression was assessed by wound-healing assay (**c**) and Matrigel Transwell assay (**d**). The data are presented as the mean ± SD. NS: not significant

To examine whether circFGFR1 promotes NSCLC cell proliferation, migration, and invasion via the miR-381-3p/CXCR4 axis, we established CXCR4-knockout NCI-H358 and NCI-H1299 cells (NCI-H358-CKO and NCI-H1299-CKO cells) through CRISPR/Cas9 (Additional file 4: Figure S7a). The CXCR4 protein did not show significant change after circFGFR1 expression was upregulated in the H358-CKO and NCI-H1299-CKO cells (Additional file 4: Figure S7b and c). Furthermore, results from the in vitro CCK-8, clone formation, wound-healing cell migration, and invasion assays revealed that the H358-CKO and NCI-H1299-CKO cells with forced circFGFR1 expression had no significantly change in malignant phenotype compared with that of the mock cells (Fig. 5a-d).

It has been reported that increased CXCR4 expression promotes epithelial-mesenchymal transition (EMT), invasion, and migration of NSCLC cells [28, 29]. Therefore, we examined EMT marker expression, including that of E-cadherin, N-cadherin, Snail, and Twist, in NSCLC cells with forced circFGFR1 expression. The results showed that circFGFR1 significantly promoted N-cadherin, Snail, and Twist expression compared with the expression in the mock groups (Additional file 4: Figure S8).

### Higher levels of circFGFR1 expression is correlated with cytotoxic T lymphocyte exclusion and resistance to anti-PD-1 NSCLC therapy

**Fig. 6:**
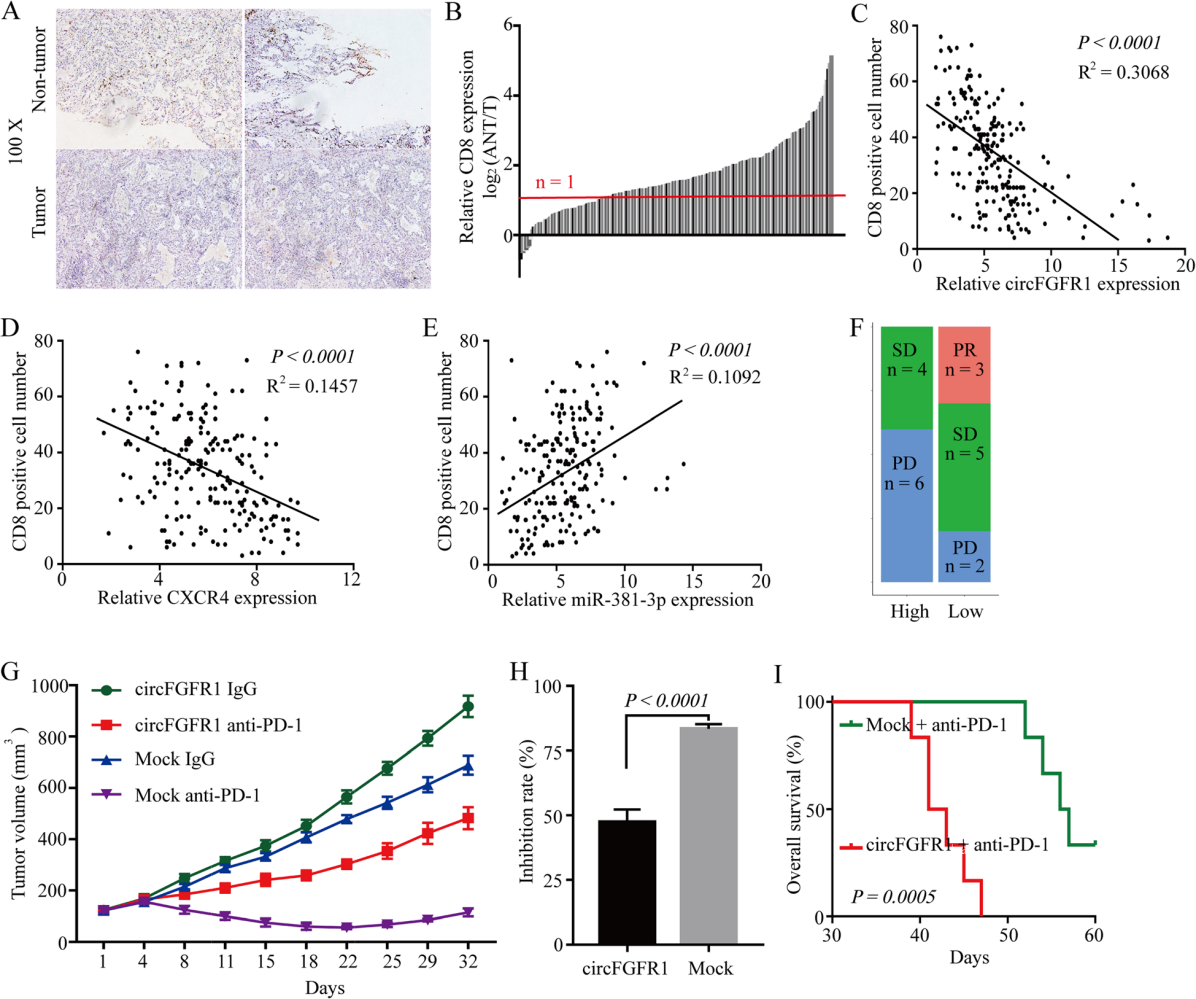
CircFGFR1 promotes immunosuppression and resistance to NSCLC immunotherapy. **a** Representative NSCLC cases in which the tissue were analyzed by IHC staining for CD8. **b** CD8+ T cells in 210 pairs of NSCLC and matched nontumor tissues, shown as log_2_ (tumor/nontumor). **c** A negative correlation between circFGFR1 and the number of CD8-positive cells was observed in the NSCLC tissues (*R*^*2*^ = 0.3068; *P* < 0.0001). **d** A negative correlation between CXCR4 and the number of CD8-positive cells was observed in the NSCLC tissues (*R*^*2*^ = 0.1457; *P* < 0.0001). **e** A positive correlation between miR-381-3p and the number of CD8-positive cells was observed in the NSCLC tissues (*R*^*2*^ = 0.1092; *P* < 0.0001). **f** Comparison of therapy efficacy for patients with high and low circFGFR1 expression and who were treated with Opdivo. **g** LLC-mock or LLC-circFGFR1 cells were subcutaneously injected into 4-week-old nude mice, and when tumors reached a mean tumor volume of 100 mm^3^, the mice were treated with an IgG or PD-1 antibody. The data are expressed as the mean tumor volume. **h** The data are expressed as the percent of tumors with inhibited growth (the data are presented as the mean ± SD). **i** Comparison of the overall survival curves for mice with high and low circFGFR1 expression of xenograft lung tumors that were treated with a mouse antibody against mouse PD-1

CXCR4 is a potential mediator that induces cytotoxic T-lymphocyte exclusion and participates in resistance to anti-PD-1 therapy of cancer [30, 31]. To further investigate the relationship between the circFGFR1/CXCR4 axis and immune escape, we measured the infiltration of CD8+ T cells in tissues from the 210 cases of NSCLC and matched nontumor tissues. The number of CD8+ T cells in the NSCLC tissues was significantly lower than that of the adjacent nontumor tissues (155/210; 2-fold) (Fig. 6a and b). The results from a scatter plot analysis revealed a negative correlation between circFGFR1/CXCR4 expression and CD8+ T cell frequency in the NSCLC tissues (Fig. 6c and d). Moreover, the results from a scatter plot analysis also revealed a positive correlation between miR-381-3p expression and CD8+ T cell frequency in the NSCLC tissues (Fig. 6e). These results suggest that circFGFR1 may exert its immunosuppressive effects by upregulating CXCR4 via sponging miR-381-3p. We then analyzed retrospective data from 20 patients with recurrent NSCLC who had undergone lung resection 2–60 months before receiving PD-1 antibody immunotherapy. After four therapy cycles, an efficacy assessment was performed using enhanced CT. In terms of RECIST1.1, the results showed that there were 3 patients with a partial response (PR), 5 patients with stable disease (SD), and 2 patients with progressive disease (PD) in the circFGFR1^low^ group; while 4 patients with stable disease (SD) and 6 patients with progressive disease (PD) were in the circFGFR1^high^ group (Fig. 6f). Overall, the above results indicated that circFGFR1 promoted NSCLC cell resistance to anti-PD-1 agents.

Interestingly, humans and mice have the same miR-381-3p sequence, and mouse CXCR4 contains a speculated seed-sequence of miR-381-3p (Additional file 4: Figure S9a). We completed a luciferase assay using miR-381-3p mimics that were cotransfected with luciferase reporters (containing mouse wild-type or the mutant miR-381-3p seed-sequence of CXCR4) into HEK-293 T cells. Compared with the negative control RNA (NC), miR-381-3p decreased the luciferase reporter activity significantly in the cells with the wild-type mouse CXCR4 sequence but not the cells with the mutant mouse CXCR4 sequence (Additional file 4: Figure S9b). Furthermore, our results showed that CXCR4 was significantly upregulated when circFGFR1 was overexpressed in LLC mouse lung cancer cells (Additional file 4: Figure S9c-e). To further assess the effects of circFGFR1 expression on anti-PD-1 therapy resistance, we analyzed the anti-tumor effects of the PD-1 antibody in xenograft C57BL/6 mice that received LLC-circFGFR1 cells or the respective mock cells. Compared to that of the mock cell group, the tumor growth in the LLC-circFGFR1 cell recipient xenograft mice showed an obvious phenotype of resistance to anti-PD-1 therapy, and the xenograft mice had a shorter survival time (Fig. 6g-i). In addition, our results showed that the expression of PD-L1 was not changed significantly when circFGFR1 was overexpressed in the LLC mouse lung cancer cells (Additional file 4: Figure S10a). Moreover, in the xenograft mice, the number of PD-1-positive LLC cells was significantly reduced when circFGFR1 was forcibly expressed (Additional file 4: Figure S10b). Consistent with the in vitro experiment, the PD-L1 expression in the LLC cells with forcibly expressed circFGFR1 was not significantly changed in the xenograft mice (Additional file 4: Figure S10c).

## Discussion

Recently, an increasing number of studies have confirmed the dysregulation of noncoding RNAs in almost all cancer types. Recent studies have verified that many miRNAs, circRNAs and lncRNAs play critical roles in modulating tumor development and progression [32, 33]. However, the mechanisms by which circRNAs participate in cancer development and progression remains unclear [34, 35]. Currently, only a few circRNAs have been verified to play a critical role in NSCLC [36, 37]. Here, we report, for the first time, that circFGFR1 was a critical circRNA that is frequently upregulated in NSCLC tissues, and high levels of circFGFR1 expression is positively correlated with poor clinicopathological characteristics, including large tumor size, lymph node metastasis, and poor cell differentiation. Furthermore, we verified that circFGFR1 acted as a sponge of miR-381-3p, thereby promoting NSCLC progression and resistance to anti-PD-1 therapy by upregulating CXCR4 expression. Overall, our results demonstrate a crucial role of circFGFR1 in NSCLC progression and response to immunotherapy.

Accumulating reported evidence indicates that circRNAs act as miRNA sponges or competitors of endogenous RNAs (ceRNAs) to induce the functional dysregulation of miRNAs and their target genes, leading to tumor proliferation, invasion, and chemo-resistance in cancers, including lung cancer [10, 38, 39]. Here, we verified that circRGFR1 promotes cell proliferation, metastasis, and immune evasion of NSCLC cells by sponging miR-318-3p. In fact, miR-318-3p has been confirmed to act as a tumor suppressor in various malignancies [40–42]. For example, miR-318-3p inhibits cervical cancer cell proliferation, migration, and invasion by targeting FGF7 [40]. Importantly, the findings from our present study are consistent with those of the majority of previous studies that show the suppressive effects of miR-318-3p on NSCLC cell proliferation, migration, invasion, and immune evasion by targeting CXCR4.

Anti-PD-1 immunotherapy has led to an effective antitumor response rate in multiple advanced cancers, including NSCLC [43, 44]. However, more than one-half of NSCLC patients do not show a long-term response to anti-PD-1-based immunotherapy [45, 46]. Recent studies have shown that dysregulation of chemokine receptor expression is one of the critical intrinsic tumor-promoting regulators in cancers, as they even promote immune system evasion [30, 47–49]. However, it was unclear how chemokine receptor expression was regulated in NSCLC cells. In this study, we found that CXCR4 expression is positively regulated by circFGFR1. Furthermore, knocking out CXCR4 sensitized NSCLC cells to anti-PD-1 immunotherapy. Similar to previous research, forced CXCL12/CXCR4 signaling in metastatic breast cancers promoted immune system evasion, making it a potential target for inhibiting the resistance of therapeutics to immune checkpoint blockades in metastatic breast cancers patients [49]. Therefore, inhibiting the CXCR4-related pathway in NSCLC cells may provide a promising opportunity to inhibit resistance to anti-PD-1 immunotherapy.

## Conclusion

Taken together, our results indicate that circFGFR1 expression is significantly increased in NSCLC tissues compared with the expression in paired adjacent nontumor tissues. Functionally and mechanistically, circFGFR1 promotes the progression and immune system evasion of NSCLC cells by sponging miR-381-3p and targeting CXCR4, which has been identified as a critical oncogene in several cancers. Therefore, inhibiting the circFGFR1/miR-381-3p/CXCR4-related pathway in NSCLC cells may hold promise for NSCLC therapy.

## Supplementary information

**Additional file 1.** Supplementary Materials and Methods.

**Additional file 2: Table S1.** The RT-qPCR primers used in this study.

**Additional file 3: Table S2.** Antibody for western blotting, RIP, and immunohistochemistry.

**Additional file 4: Figure S1.** CircFGFR1 expression in the NSCLC cells. **Figure S2.** Effects of decreased circFGFR1 expression on the progression of the NSCLC cells. **Figure S3.** The relationship between circFGFR1 and miR-381-3p expression in the NSCLC cells. **Figure S4.** The predicted target mRNAs of miR-381-3p were identified in vivo. **Figure S5.** miR-381-3p inhibited CXCR4 expression in the NSCLC cells. **Figure S6.** The levels of CXCR4 and miR-381-3p in the NSCLC tissues and prognostic significance. **Figure S7.** Knocking out CXCR4 in NSCLC cells via CRISPR/Cas9 technology. **Figure S8.** E-cadherin, N-cadherin, Twist, and Snail protein expression levels in the NCI-H358 and NCI-H1299 cells was modified by circFGFR1 transfection. **Figure S9.** CXCR4 binds to miR-381-3p in the mouse NSCLC cells. **Figure S10.** Effects of forced circFGFR1 expression on the immune inhibition of NSCLC cells.

## Data Availability

All data generated or analyzed during this study are included either in this article or in the supplementary information files.

## Abbreviations

CCK-8: Cell Counting Kit-8
CircRNA: Circular RNA
CXCR4: C-X-C motif chemokine receptor 4
EMT: Epithelial-mesenchymal transition
FGFR1: Fibroblast growth factor receptor 1
HPV: Human papillomavirus
IHC: Immunohistochemistry
NSCLC: Non-small-cell lung cancer
PD-1: Programmed cell death 1
RT-qPCR: Quantitative real-time polymerase chain reaction
TET1: Tet methylcytosine dioxygenase 1
TMA: Tissue microarray
