# Correction to: Circular RNA circFGFR1 promotes progression and anti-PD-1 resistance by sponging miR-381-3p in non-small cell lung cancer cells

**DOI:** 10.1186/s12943-020-1131-y

**Published:** 2020-02-01

**Authors:** Peng-Fei Zhang, Xu Pei, Ke-Sang Li, Li-Na Jin, Fei Wang, Jing Wu, Xue-Mei Zhang

**Affiliations:** 1 Department of Oncology, Shanghai East Hospital, Tongji University School of Medicine, Shanghai, China; 2 Department of Cardiothoracic Surgery, the Second Affiliated Hospital of Nanchang University, Nanchang, Jiangxi China; 3 Department of Hematology and Oncology, Hwa Mei Hospital, University of Chinese Academy of Sciences, Zrhejiang, Ningbo, China; 4 Department of Hematology, Shanghai East Hospital, Tongji University School of Medicine, Shanghai, China

**Correction to: Mol Cancer**

**https://doi.org/10.1186/s12943-019-1111-2**

After the publication of this work [1], an error was found in Fig. 1b. As described in the Results section that circ0084003 has 13 exons, it should be formed by 5–17 (13exons) exons, authors have described “formed by 5-19 exons”. The authors extend their apology for the mistake caused by their action. With that mistake, the correct version of Fig. 1b is provided below.


