# Supplementary material for: Circular RNA circFGFR1 promotes progression and anti-PD-1 resistance by sponging miR-381-3p in non-small cell lung cancer cells
Source: Mol Cancer. 2019 Dec 9;18:179. doi: 10.1186/s12943-019-1111-2 (PMC6900862; doi:10.1186/s12943-019-1111-2)
Supplement: Supplementary file 1 — Additional file 1. Supplementary Materials and Methods. [file 12943_2019_1111_MOESM1_ESM.docx]

**Supplementary Materials and Methods**

***RT-qPCR***

Total RNA was extracted using TRIzol reagent (Sigma, St. Louis, MO, USA) according to the manufacturer’s instructions. RT-qPCR was performed using an ABI PRISM 7900 sequence detection system (Applied Biosystems, Foster City, CA, USA) using a SYBR Green Real-Time PCR Master Mix kit (TaKaRa, Japan). Expression levels of the circRNAs and CXCR4 were normalized to those of glyceraldehyde 3-phosphate dehydrogenase (GAPDH). U6 snRNA was used as a reference for miRNA-381-3p. Each sample was tested in triplicate. The relative expression was analyzed by the comparative cycle threshold (Ct) method according to the equation 2-ΔCt [ΔCt = Ct-Ct (GAPDH)].

***Western blotting***

The total protein extracted from cells was separated by sodium dodecyl sulfate-polyacrylamide gel electrophoresis (SDS-PAGE), transferred onto polyvinylidene difluoride membranes, and incubated with the corresponding antibodies. The membranes were developed using the enhanced chemiluminescence method (Pierce, Rockford, IL, USA).

***IHC assays and correlations between two groups***

In brief, antibodies against PD-L1, PD-1, CXCR4 or CD8 were used to detect the expression of CXCR4 or CD8. A positive reaction for CXCR4/CD8 was scored in one of four grade categories depending on the intensity of the staining, i.e., 0, 1, 2 or 3, and the percentage of CXCR4/CD8-positive cells was also scored based on one of four categories: 0 (0%), 1 (1 to 33%), 2 (34 to 66%) and 3 (67 to 100%). In cases with discrepancies between the duplicated cores, the higher score of the two tissues was taken as the definitive score. The sum of the intensity and percentage scores was used as the final staining score. Then, the correlations between the two groups were determined based on the Pearson’s correlation coefficient.

***Fluorescence in situ hybridization (FISH)***

Cy3-labeled probes specific to circFGFR1 and fam-labeled probes specific to miR-381-3p were generated. The probes were designed and synthesized by GenePharma (Shanghai, China), and the signals of the probes were detected by a fluorescent in situ hybridization kit (GenePharma, Shanghai, China) according to the manufacturer’s instructions. The images were taken with a fluorescence microscope (Leica Microsystems Imaging Solutions, Cambridge, UK).

***Cell proliferation, clonal formation, wound-healing migration, and Matrigel invasion assays***

Cell proliferation was detected by the Cell Counting Kit-8 (CCK-8, Yeasen, Shanghai, China) Kit. In brief, cells were inoculated into 96-well plates (1, 000 cells per well). Then, 10 μl of CCK-8 reagent was added to the wells after the 1st, 2nd, 3rd, 4th, and 5th days. The plates were incubated for 2 h, and the absorbance was determined at 450 nm. For colony formation assay, NSCLC cells were seeded in a 6 cm culture dish (1, 000 cells. After 14 days, the cells were washed with PBS, fixed with 4% paraformaldehyde, and stained with 0.4% crystal violet for 15 min. For wound-healing migration assays, the cell monolayers were mechanically disrupted using a sterile 200-μl pipette tip to generate a linear wound. The average distance migrated by the cells was measured using a microscope calibrated with an ocular micrometer at a suitable time. For invasion assays, cells were incubated using 24-well transwell plates (8-μm pore size, Corning, NY, USA). One million cells suspended in serum-free medium were plated in the upper chambers with Matrigel (BD Biosciences, USA), and 0.6 ml of DMEM or RPMI-1640 medium with 10% FBS was added to the lower chamber. After incubation for a suitable amount of time, the cells were fixed in 4% paraformaldehyde, stained by crystal violet, and counted under a microscope.

***In vivo tumor growth and metastasis assays***

Xenograft experiments in nude mice were approved by the Animal Experimentation Ethics Committee of East Hospital, Tongji University. Male BALB/c nude mice aged 4–6 weeks were maintained according to the stated guidelines. For in vivo tumor growth assay, NSCLC cells (2×10^6^) were injected into the right flank to generate subcutaneous tumors. Tumor size was measured every four days, and tumor volume was calculated as (length x width^2^)/2. Thirty days after injection, the tumor specimens were surgically removed, fixed, paraffin-embedded, and sectioned. The sections were used for H&E and IHC staining. For metastasis assay, 2×10^5^ cells (per mouse) were resuspended in 100 μl of PBS and injected them into the lateral tail vein. The mice were sacrificed after 30 days; the lungs were resected, embedded in paraffin, and stained with hematoxylin and eosin (H&E), and lung metastases were counted.

***In vivo PD-L1 and PD-1 expression assays***

Experiments in Xenograft C57BL/6 mice were approved by the Animal Experimentation Ethics Committee of East Hospital, Tongji University. Male C57BL/6 mice aged 4–6 weeks were maintained according to the stated guidelines. LLC cells (2×10^6^) with or without forced circFGFR1 were injected into the right flank to generate subcutaneous tumors. Twenty-eight days after the injection, the tumor specimens were surgically removed, fixed, embedded in paraffin, and cut into sections.

***Statistical analysis***

Statistical analysis was performed with SPSS software (19.0; SPSS, Inc., Chicago, IL). In brief, the values are expressed as the mean ± standard deviation (SD). Student’s t test was used for comparisons between groups. The categorical data were analyzed by chi-square or Fisher’s exact tests. Correlation analysis was performed among circFGFR1, miR-381-3p, CXCL4, and CD8. The cumulative recurrence and survival rates were analyzed using Kaplan-Meier’s method and the log-rank test. Cox’s proportional hazard regression model was used to analyze independent prognostic factors. P< 0.05 was considered statistically significant.
