# Supplementary material for: Circular RNA circFGFR1 promotes progression and anti-PD-1 resistance by sponging miR-381-3p in non-small cell lung cancer cells
Source: Mol Cancer. 2019 Dec 9;18:179. doi: 10.1186/s12943-019-1111-2 (PMC6900862; doi:10.1186/s12943-019-1111-2)
Supplement: Supplementary file 2 — Additional file 2: Table S1. The RT-qPCR primers used in this study. [file 12943_2019_1111_MOESM2_ESM.docx]

Table S1. The RT-qPCR primers used in this study.

| **Gene** | **Forward primer (5’-3’)** | **Reverse primer(5’-3’)** |
| --- | --- | --- |
| Circ_0084000 | CCTTGACCTCCAACCAGGTC | TTGGAGGCCAGATACTCCATG |
| Circ_0084001 | CCTTGACCTCCAACCAGATAC | ATCTGGACATAAGGCAGGTTG |
| Circ_0084002 | GCCTTGACCTCCAACCAGGTC | ACATGAACTCCACGTTGCTAC |
| Circ_0084003 | GCCTTGACCTCCAACCAGCCG | GTGGCATAACGGACCTTGTAG |
| Circ_0084004 | GCCTTGACCTCCAACCAGAAG | GGATACTCCACAGTGAGCTCG |
| Circ_0084005 | GTATCTGGCCTCCAAGAAGGG | GGAAGGACTCCACTTCCACAG |
| Circ_0084006 | GGTTGACCGTTCTGGAAGGTC | TGCTTTAGCCACTGGATGTGC |
| Circ_0084007 | TTATGTCCAGATCTTGAAGGG | GGAAGGACTCCACTTCCACAG |
| Circ_0084008 | ACCAGCTGGATGTCGTGGCCC | GAGAAGTAGGTGGTGTCACTG |
| Circ_0084009 | GAATTGGAGGCTACAAGGGTC | GCTCTGCACATCGTCCCGCAG |
| Circ_0084010 | GGTCCTGCCAGCCGAAGGGTC | TGCCATCCTTGTTCAGGCAAG |
| Circ_0136505 | TGGTTGACCGTTCTGGAAGCCC | GTCCTGCACCTCCACCTCCTC |
| Circ_0002352 | CCGACCTTGCCTGAACAAGGG | GGACGGCCTAGCGGTGCAGAG |
| Circ_0005564 | TATGTCCAGATCTTGAAGGTC | ACATGAACTCCACGTTGCTAC |
| Circ_0008016 | TCTCCGTCAATGTTTCAGCCC | GGAGTCCTGCACCTCCACCTC |
| Circ_0083998 | CCTTGACCTCCAACCAGGGCC | TGACCCTCCTTCAGCAGCTTG |
| Circ_0083999 | GCCTTGACCTCCAACCAGTGC | CACTCTGGTGGGTGTAGATCC |
| CXCR4 | CCACGCCACCAACAGTCAGAG | CTTCTGGTGGCCCTTGGAGTG |
| GAPDH | GGGGCTCTCCAGAACATCATCC | ACGCCTGCTTCACCACCTCTT |
