# Supplementary material for: Circular RNA circFGFR1 promotes progression and anti-PD-1 resistance by sponging miR-381-3p in non-small cell lung cancer cells
Source: Mol Cancer. 2019 Dec 9;18:179. doi: 10.1186/s12943-019-1111-2 (PMC6900862; doi:10.1186/s12943-019-1111-2)
Supplement: Supplementary file 3 — Additional file 3: Table S2. Antibody for western blotting, RIP, and immunohistochemistry. [file 12943_2019_1111_MOESM3_ESM.docx]

Table S2. Antibody for western blotting, RIP, and immunohistochemistry.

| **Antibody** | **Company** | **Cat No.** |
| --- | --- | --- |
| CXCR4 | Abcam | Ab181020 |
| β-actin | Abcam | ab8226 |
| CD8 | Abcam | ab4055 |
| PD-L1 | Abcam | ab213480 |
| PD-1 | Abcam | ab214421 |
| HRP-labeled Goat Anti-Rabbit IgG(H+L) | Beyotime | A0208 |
| HRP-labeled Goat Anti-mouse IgG(H+L) | Beyotime | A0216 |
| AGO2 | Abcam | ab32381 |
| IgG | Abcam | ab172730 |
