# Supplementary material for: Circular RNA circFGFR1 promotes progression and anti-PD-1 resistance by sponging miR-381-3p in non-small cell lung cancer cells
Source: Mol Cancer. 2019 Dec 9;18:179. doi: 10.1186/s12943-019-1111-2 (PMC6900862; doi:10.1186/s12943-019-1111-2)
Supplement: Supplementary file 4 — Additional file 4: Figure S1. CircFGFR1 expression in the NSCLC cells. Figure S2. Effects of decreased circFGFR1 expression on the progression of the NSCLC cells. Figure S3. The relationship between circFGFR1 and miR-381-3p expression in the NSCLC cells. Figure S4. The predicted target mRNAs of miR-381-3p were identified in vivo. Figure S5. miR-381-3p inhibited CXCR4 expression in the NSCLC cells. Figure S6. The levels of CXCR4 and miR-381-3p in the NSCLC tissues and prognostic significance. Figure S7. Knocking out CXCR4 in NSCLC cells via CRISPR/Cas9 technology. Figure S8. E-cadherin, N-cadherin, Twist, and Snail protein expression levels in the NCI-H358 and NCI-H1299 cells was modified by circFGFR1 transfection. Figure S9. CXCR4 binds to miR-381-3p in the mouse NSCLC cells. Figure S10. Effects of forced circFGFR1 expression on the immune inhibition of NSCLC cells. [file 12943_2019_1111_MOESM4_ESM.docx]

**Figure S1**


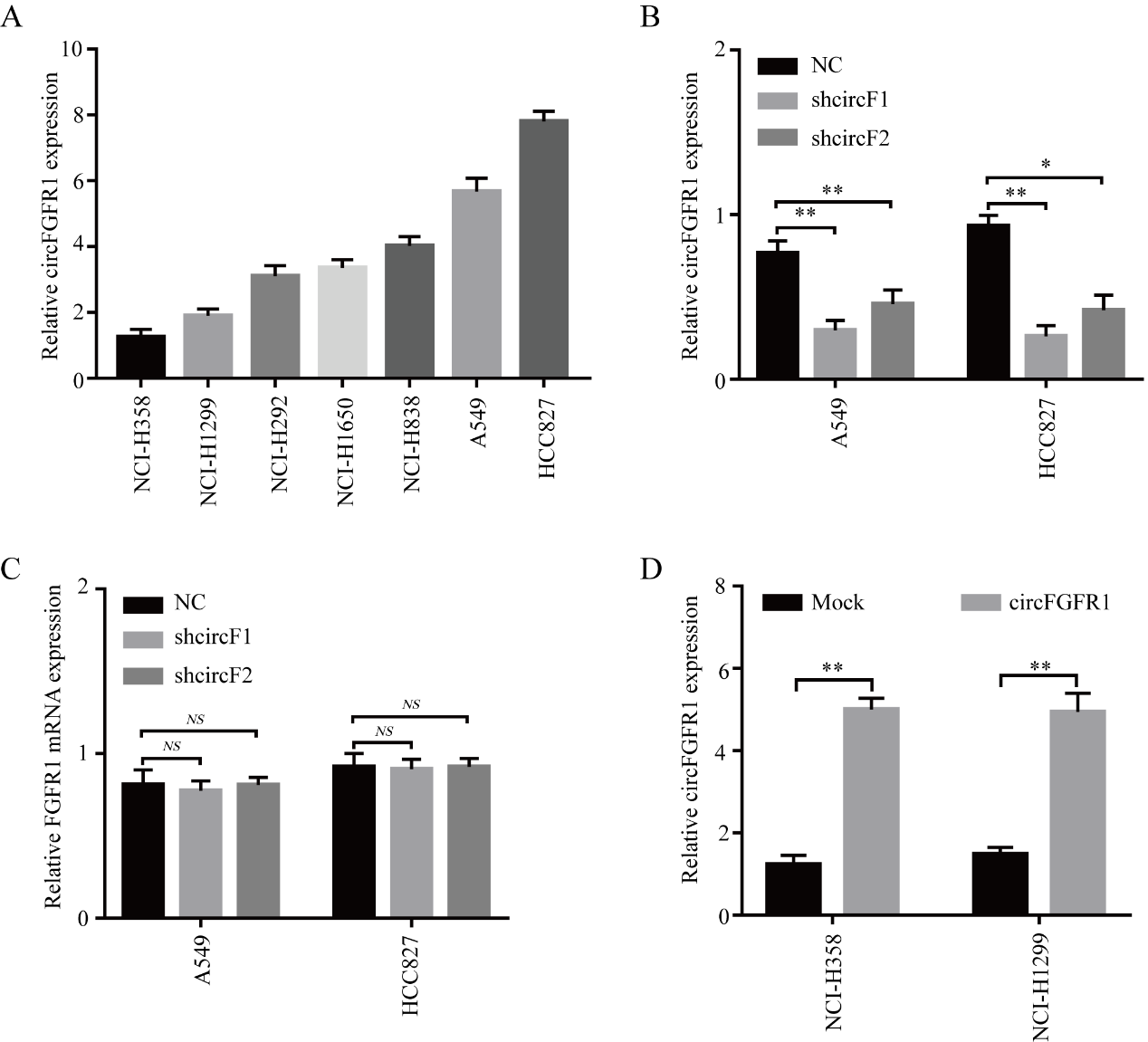


**Figure S1. CircFGFR1 expression in the NSCLC cells.** **a** circFGFR1 expression in several NSCLC cell lines was measured using RT-qPCR analysis. **b** circFGFR1 expression in the A549 and HCC827 cells was modified by transfection of shRNA to cause interference. **c** FGFR1 mRNA expression in circFGFR1-knockdown NSCLC cells. **d** circFGFR1 expression in the NCI-H358 and NCI-H1299 cells was modified by cDNA transfection. The data are presented as the mean ± SD, n=3. *P < 0.05; **P < 0.01; NS, not significant.

**Figure S2**


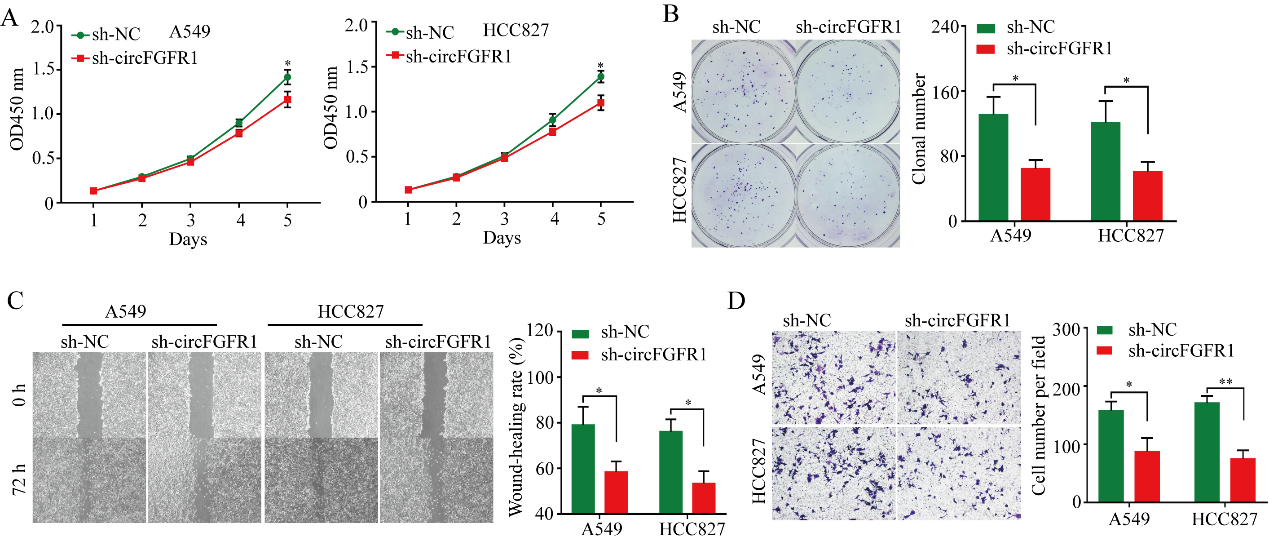


**Figure S2. Effects of decreased circFGFR1 expression on the progression of the NSCLC cells**. **a** and **b** Proliferation in NSCLC cells with decreased expression of circFGFR1 was assessed by CCK-8 assay (a) and clonal formation assay (b). **c** and **d** Migration and invasion of the NSCLC cells with decreased circFGFR1 expression were assessed by wound healing assay (c) and Matrigel Transwell assay (d). The data are presented as the mean ± SD, *P < 0.05, **P < 0.01.

**Figure S3**


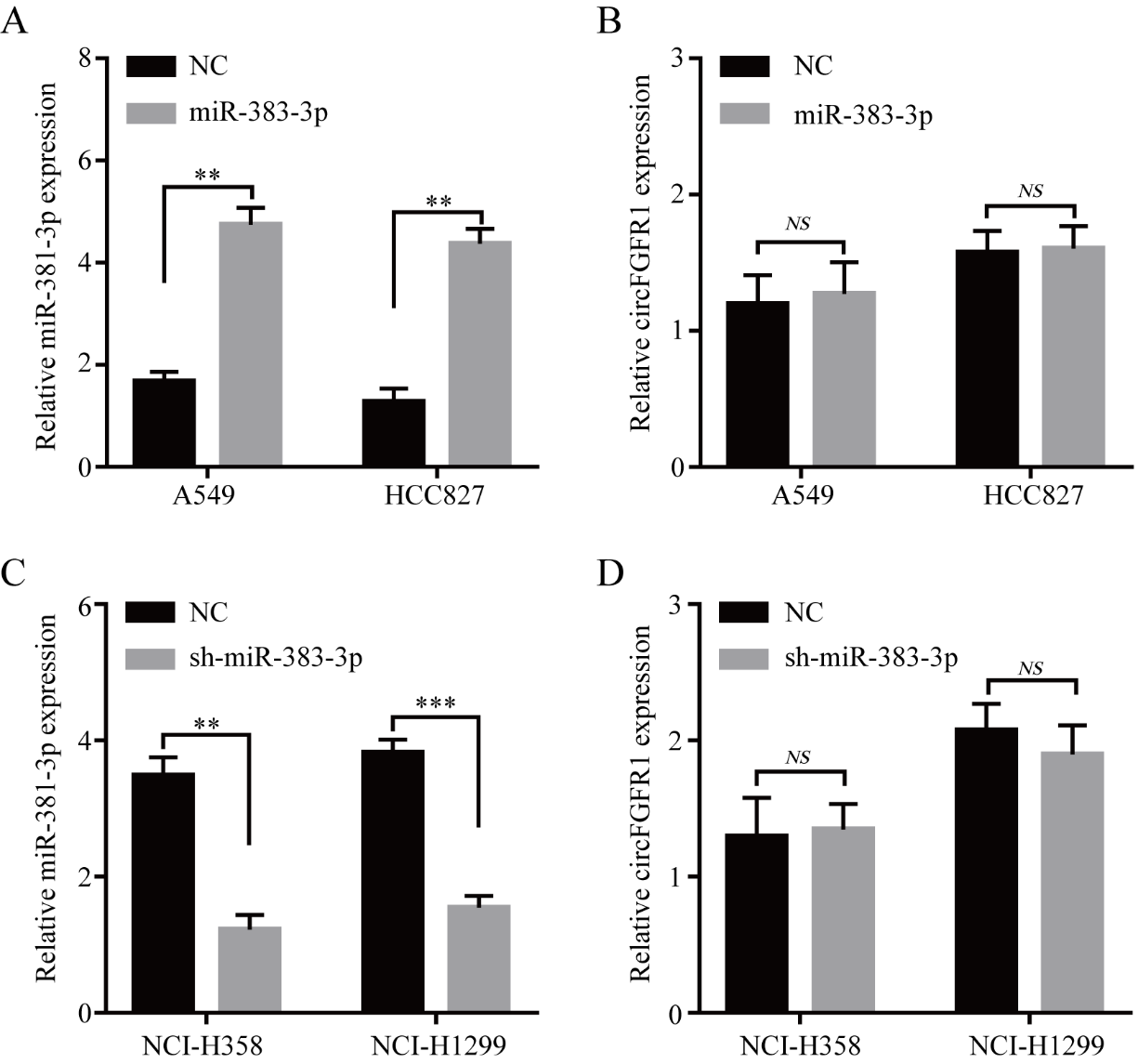


**Figure S3. The relationship between circFGFR1 and miR-381-3p expression in the NSCLC cells**. **a** miR-381-3p expression in the A549 and HCC827 cells was modified by cDNA transfection. **b** circFGFR1 expression in the miR-381-3p-overexpressing NSCLC cells. **c** miR-381-3p expression in the A549 and HCC827 cells was modified by shRNA transfection. **d** circFGFR1 expression in the miR-381-3p-knockdown NSCLC cells. The data are presented as the mean ± SD, n=3. **P < 0.01; ***P < 0.001; NS, not significant.

**Figure S4**

**
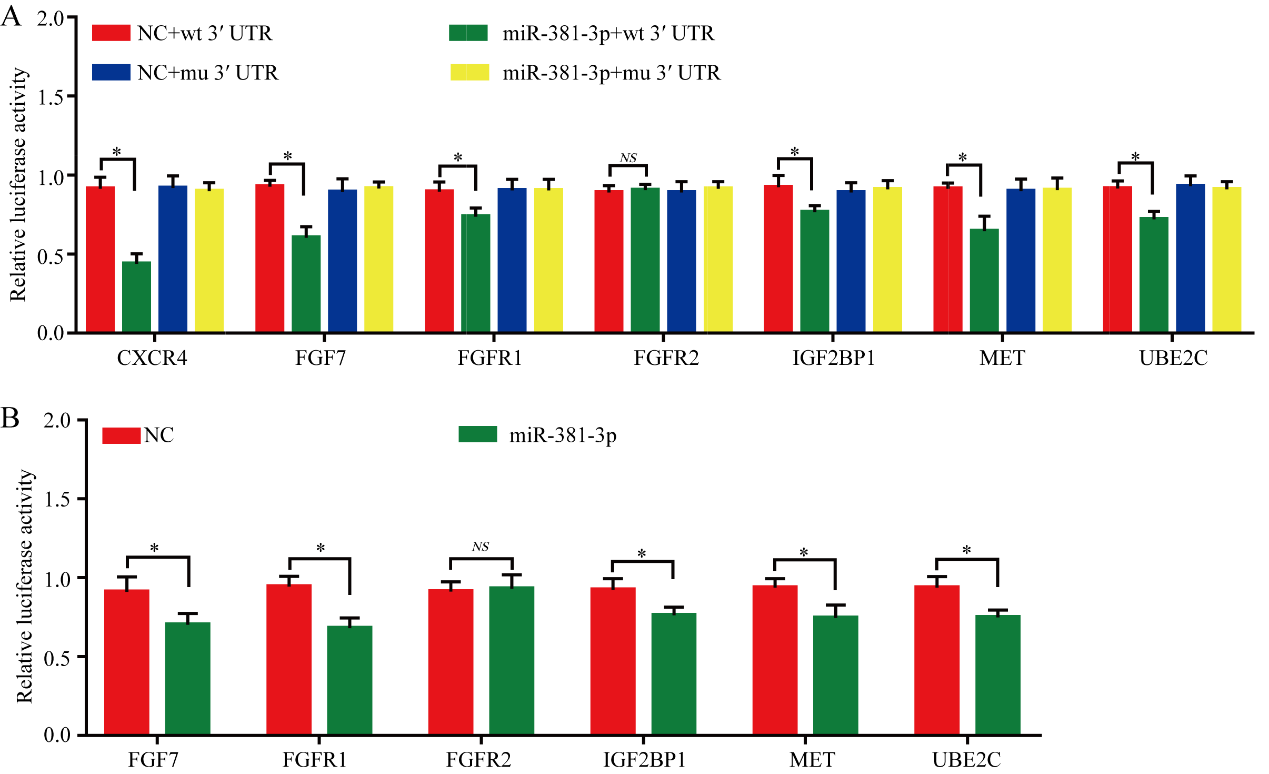
**

**Figure S4. The predicted target mRNAs of miR-381-3p were identified in vivo. a** The luciferase activity of wild-type LUC-CXCR4/FGF7/FGFR2/FGFR1/IGF2BP1/UBE2C or mutant LUC-CXCR4/FGF7/FGFR2/FGFR1/IGF2BP1/UBE2C in the HEK-293T cells cotransfected with miR-191 or the negative control (NC). **b** FGF7, FGFR2, FGFR1, IGF2BP1, and UBE2C mRNA expression in the A549 cells was changed by miR-381-3p siRNA transfection. The data are presented as the mean ± SD, n=3. *P < 0.05; NS, not significant.

**Figure S5**


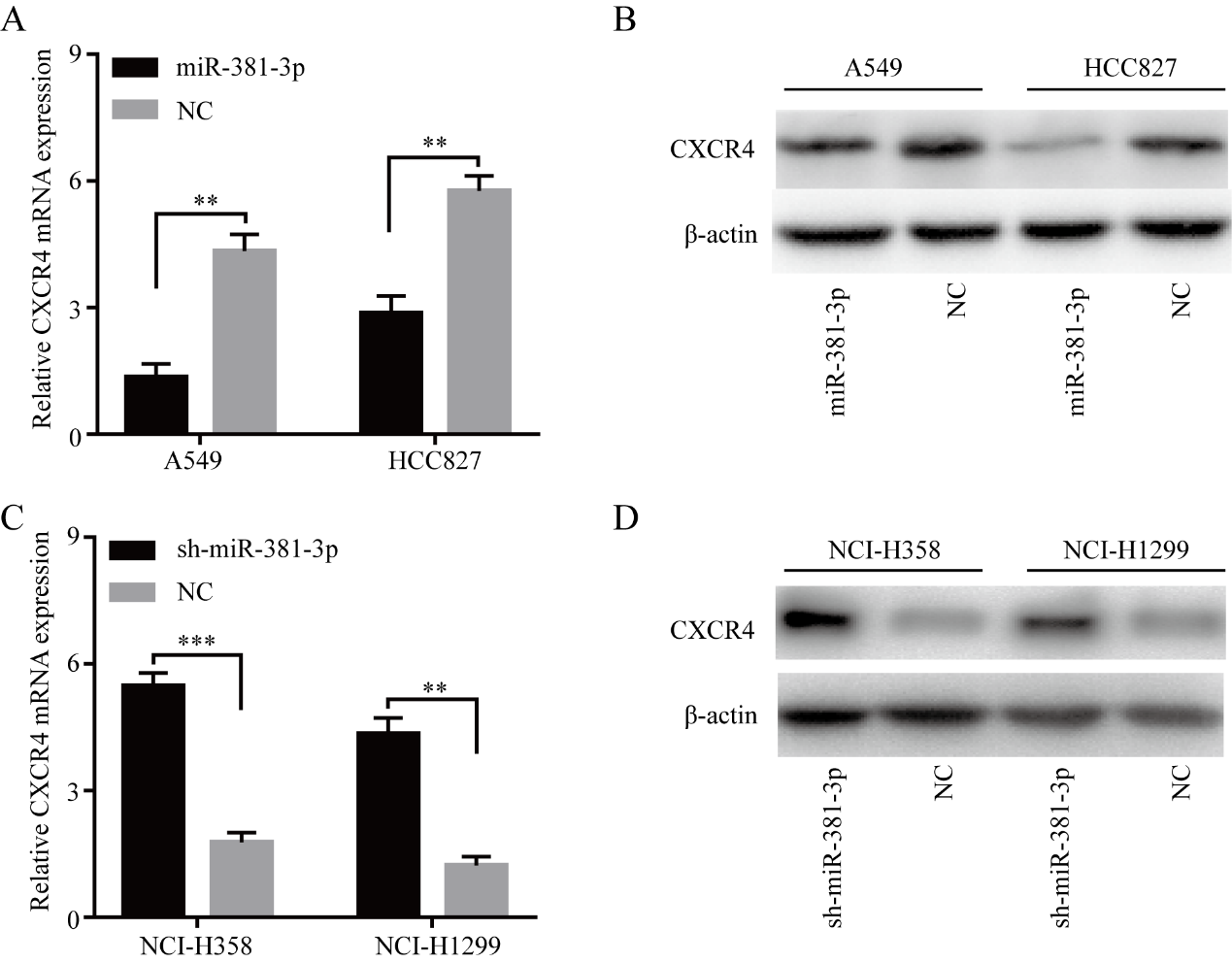


**Figure S5. miR-381-3p inhibited CXCR4 expression in the NSCLC cells.** **a** and **b** CXCR4 mRNA and protein expression in miR-381-3p-overexpressing NSCLC cells. **c** and **d** CXCR4 mRNA and protein expression in miR-381-3p-knockdown NSCLC cells. The data are presented as the mean ± SD, n=3. **P < 0.01; ***P < 0.001.

**Figure S6**

**
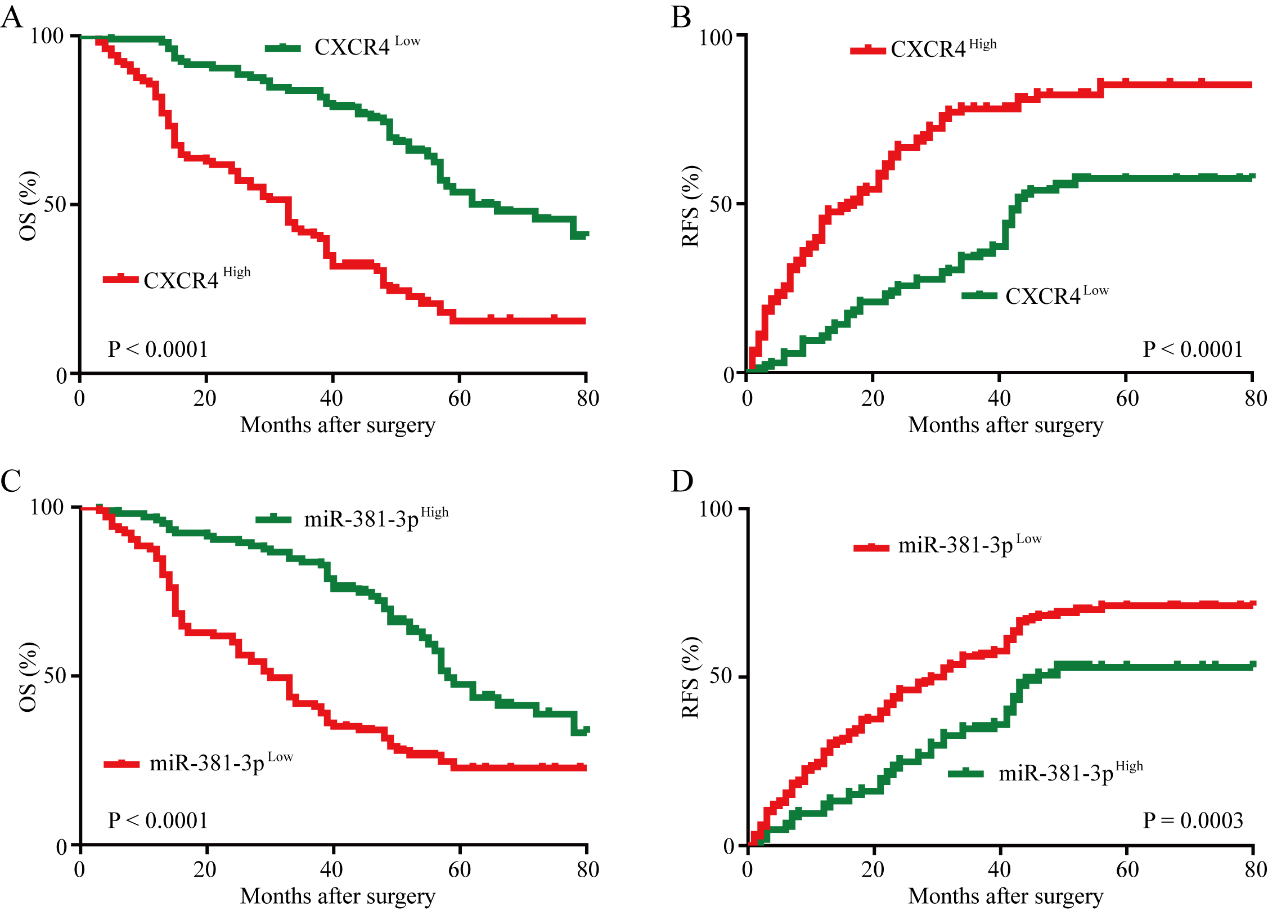
**

**Figure S6. The levels of CXCR4 and miR-381-3p in the NSCLC tissues and prognostic significance**. **a** and **b** Prognostic analysis of CXCR4 expression in 210 NSCLC patient tissues. **c** and **d** Prognostic analysis of miR-381-3p expression in 210 NSCLC patient tissues.

**Figure S7**


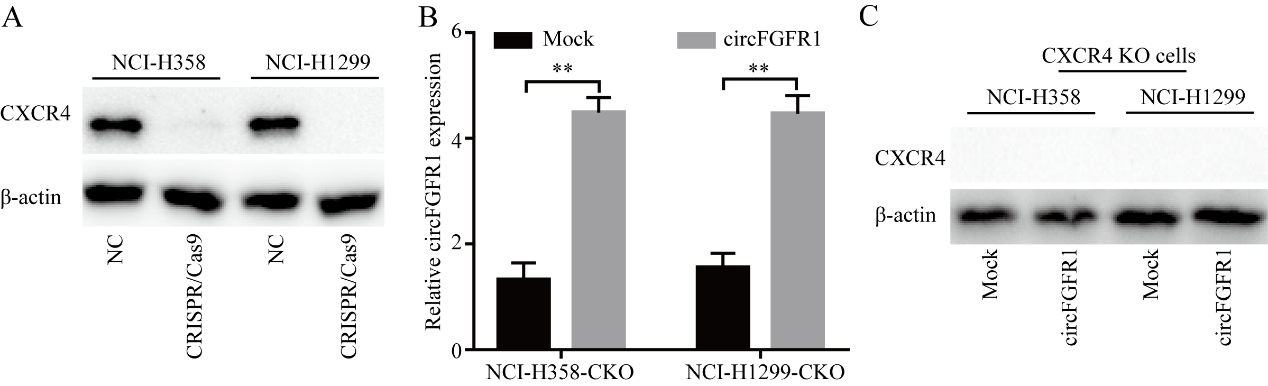


**Figure S7. Knocking out CXCR4 in NSCLC cells via CRISPR/Cas9 technology. a** CXCR4 expression in the NSCLC cell lines was examined using western blotting analysis. **b** circFGFR1 expression in the NCI-H358-CKO and NCI-H1299-CKO cells was modified by cDNA transfection. **c** CXCR4 protein expression in the circFGFR1-overexpressing NSCLC-CKO cells. The data are presented as the mean ± SD, n=3. **P < 0.01. CKO: CXCR4 knockout.

**Figure S8**

**
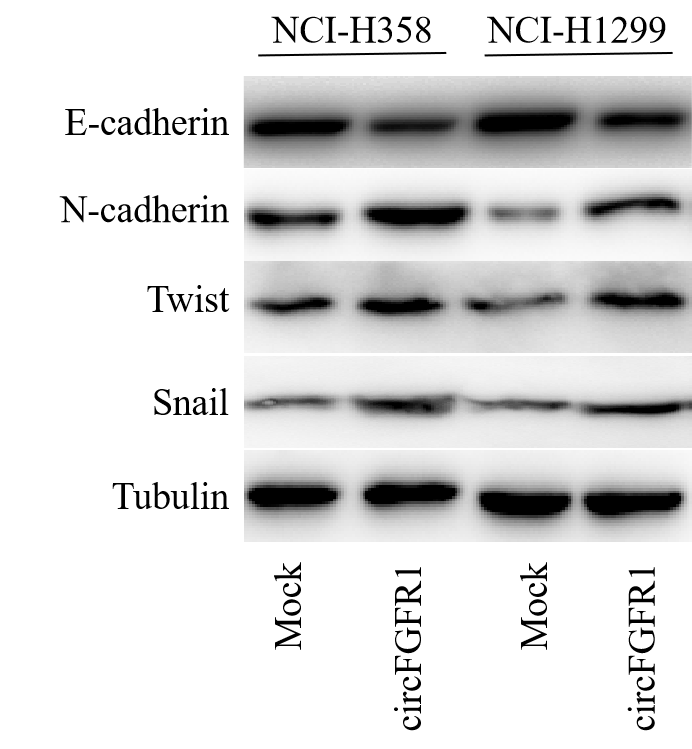
**

**Figure S8.** E-cadherin, N-cadherin, Twist, and Snail protein expression levels in the NCI-H358 and NCI-H1299 cells was modified by circFGFR1 transfection.

**Figure S9**


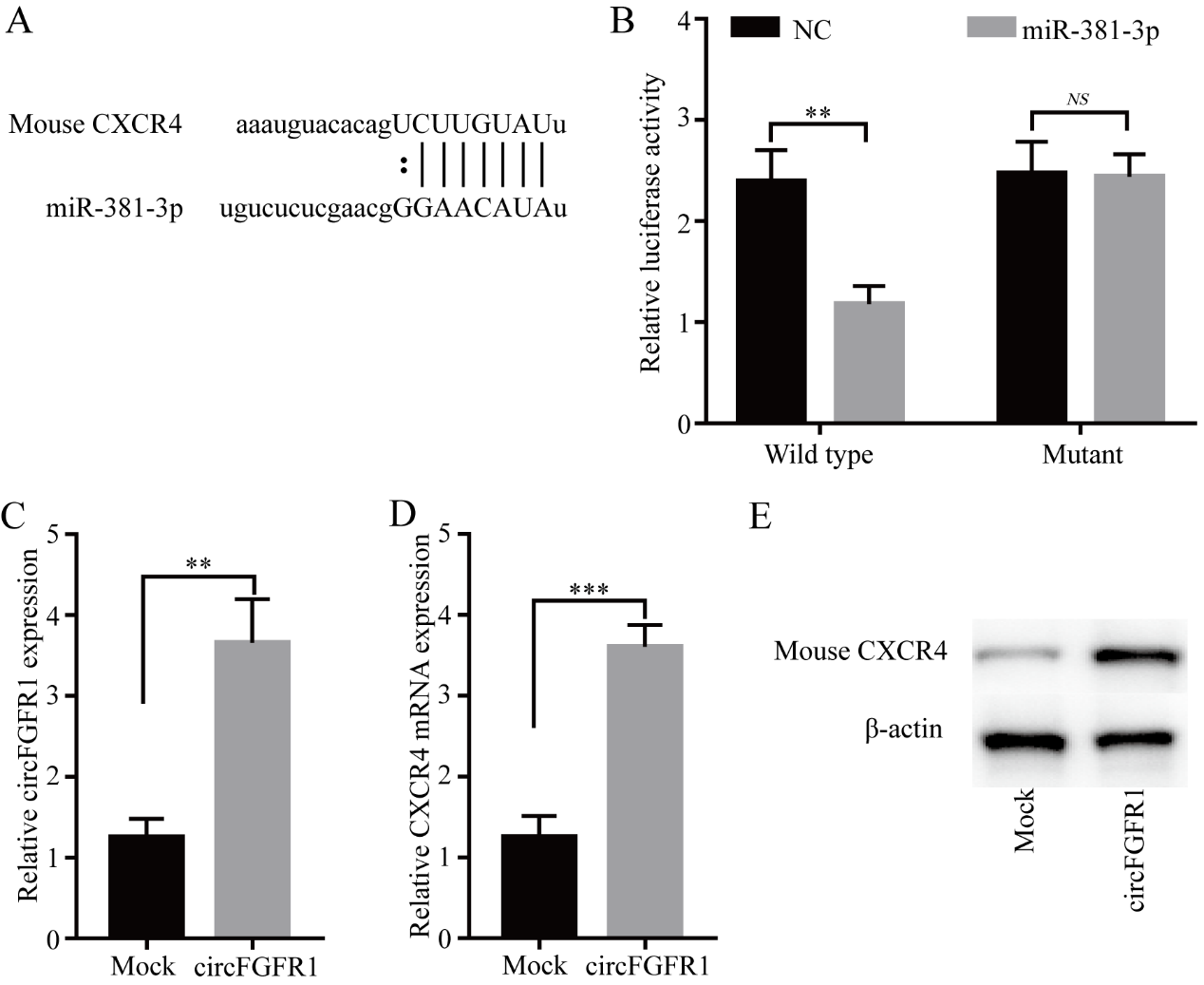


**Figure S9. CXCR4 binds to miR-381-3p in the mouse NSCLC cells.** a Putative binding sites of miR-381-3p with respect to mouse CXCR4 were predicated via StarBase v3.0. **b** The luciferase activity of pLG3-mouse CXCR4 in the HEK-293T cells cotransfected with miR-381-3p. **c** Level of circFGFR1 expression in the mouse LLC cells was modified by cDNA transfection. **d** and **e** The levels of mouse CXCR4 mRNA and protein expression were measured using RT-qPCR or western blotting in the NSCLC transfected with circFGFR1 or the control. The data are presented as the mean ± SD, n=3. **P < 0.01; ***P < 0.001; NS, not significant.

**Figure S10**


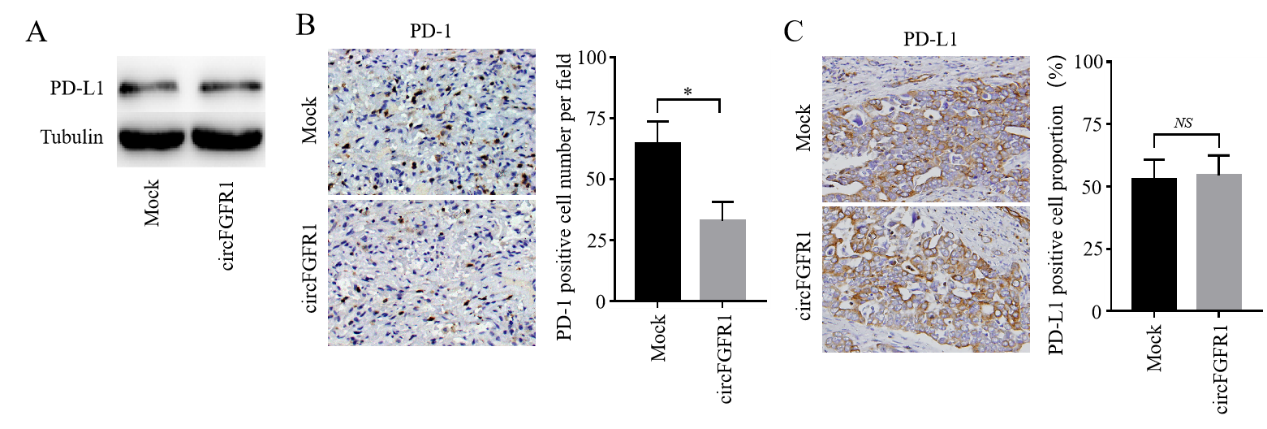


**Figure S10. Effects of forced circFGFR1 expression on the immune inhibition of NSCLC cells**. a The levels of PD-L1 was detected in LLC cells after transfection with circFGFR1 or the control using Western blotting. **b** PD-1 positive cells in the LLC-circFGFR1 or LLC-Mock cells derived tissues were analyzed by IHC. **c** PD-L1 positive cells in the LLC-circFGFR1 or LLC-Mock cells derived tissues were analyzed by IHC. The data are represented as the mean ± SD, n=6. *P < 0.05; NS, no significant.
